# ﻿Evidence for moth pollination in a rhinomyiophilous Erica species from the Cape Floristic Region of South Africa

**DOI:** 10.3897/phytokeys.246.126310

**Published:** 2024-09-02

**Authors:** Timotheüs van der Niet, Ruth J. Cozien

**Affiliations:** 1 Centre for Functional Biodiversity, School of Life Sciences, University of KwaZulu-Natal, Pietermaritzburg, 3209, South Africa University of KwaZulu-Natal Pietermaritzburg South Africa

**Keywords:** Colour, *Ericacylindrica*, *Ericainfundibuliformis*, flower orientation, hawkmoth, long-proboscid fly, moth-pollination, scent

## Abstract

Contrasting pollination syndromes in closely related species suggest that floral trait divergence is associated with differences in pollination system, but empirical observations are required to confirm syndrome-based predictions. We present a comparative study of two closely related *Erica* species with contrasting pollination syndromes from the Cape Floristic Region of South Africa. *Ericacylindrica* has narrowly tubular pale and strongly scented flowers and is known to be hawkmoth-pollinated. The closely related *Ericainfundibuliformis* has bright flower colours and appears to lack scent, traits that are suggestive of pollination by long-tongued nemestrinid flies (rhinomyiophily). Floral trait measurements revealed that both species exhibit predominantly upright flower orientation and elongated floral tubes, although tube length of *E.infundibuliformis* is consistently greater than that of *E.cylindrica*. For both species, petals are brighter than floral tube surfaces, but flowers of *E.cylindrica* lack the strong UV reflectance found in *E.infundibuliformis.* Nectar of *E.infundibuliformis* is more concentrated and produced in larger volumes. Scent composition, but not evening scent emission rates, differed between the species: scent of *E.cylindrica* is dominated by aromatic compounds, whereas scent of *E.infundibuliformis* is dominated by (E)-ocimene and other terpenoid compounds and is emitted at higher rates during the day than the evening. Pollinator observations contradicted trait-based predictions: although a single nemestrinid fly captured in the vicinity of *E.infundibuliformis* did carry *Erica* pollen, almost all other diurnal flower visitors were nectar-robbing Hymenoptera which did not carry *Erica* pollen. Contrary to predictions, at two sites and over two flowering seasons, flowers were consistently visited in the evenings by several species of settling moths and hawkmoths which carried pollen, almost exclusively of *Erica*, on their proboscides. Our findings thus suggest that, despite objective differences in key floral traits between the closely related hawkmoth-pollinated *E.cylindrica* and *E.infundibuliformis*, moths are also important pollinators of *E.infundibuliformis*. A bimodal pollination system involving predominant pollination by moths and occasional visits by long-proboscid flies could partially reconcile findings with predictions. Our study further suggests that hawkmoth pollination may be more widespread in both *Erica* and the broader Cape flora than has hitherto been assumed and emphasises the importance of nocturnal pollinator observations.

## ﻿Introduction

There is strong evidence that pollinators have been important drivers of the radiation of angiosperms, especially in lineages in which interspecific variation in suites of floral traits is associated with variation in functional pollinator groups (e.g. Grant and Grant (1965); Johnson et al. (1998); Goldblatt and Manning (2006); Whittall and Hodges (2007)). Such covariation often leads to recognition of particular suites of floral traits as ‘pollination syndromes’ (Faegri and van der Pijl 1979; Fenster et al. 2004). Pollination syndromes can be used in a predictive framework: based on a subset of species for which both floral traits and pollination systems have been documented, pollinators can be predicted for those species lacking pollinator observations. This approach has been utilised successfully for pollination systems characterised by high degrees of specialisation (Johnson and Steiner 2000; Rosas-Guerrero et al. 2014; Dellinger 2020), both for particular pollination guilds (Pauw 2006, 2022; Valverde-Espinoza et al. 2021;) and across an entire flora (Johnson and Wester 2017). Confirmation of syndrome-based predictions strengthens the evidence for associations between particular floral traits and specific pollinator groups. This information, in turn, provides better understanding of the ecology and sensory perception of functional pollinator groups (Schiestl and Johnson 2013). However, instances in which empirical evidence contradicts predictions also advance our understanding of evolutionary ecology. Firstly, falsification of syndrome-based predictions has revealed that pollination systems in some plant species may not be as specialised as suggested by pollination syndrome theory (Ollerton 1996; Waser et al. 1996). Secondly, observations that contradict syndrome-based predictions have generated useful insight into the ecological context in which specialised interactions implied by syndromes break down (e.g. de Merxem et al. (2009)). Finally, mismatches between observations and syndrome-based predictions shed light on differences between human and pollinator sensory perception (Cozien et al. 2019; Castañeda-Zárate et al. 2021), emphasising the importance of objective approaches for quantification and interpretation of floral traits, especially those such as floral scent and colour for which human perception is particularly biased (Leonardos et al. 1969; Kevan et al. 1996). Therefore, empirical tests of predictions derived from pollination syndromes can provide useful insights into plant-pollinator interactions and function of floral traits.

In plant groups for which phylogenetic relationships have been reconstructed, syndrome-based predictions of pollination systems are particularly useful for investigating potential pollinator-driven divergence between closely-related species with contrasting pollination syndromes. Differences in floral syndromes suggest that divergence is potentially driven by adaptation to different pollinators (Johnson 2006; Van der Niet et al. 2014a). Such pollinator shifts often occur along ‘lines of least resistance’ (Stebbins 1970; Johnson et al. 1998), potentially involving only minor divergence in few key traits. Investigation of phenotypic differences between closely-related taxa that differ in pollination system can thus be used to identify which key traits underpin pollinator shifts (Goldblatt et al. 2004; Shuttleworth and Johnson 2009; Castañeda-Zárate et al. 2021). However, floral divergence may not necessarily be accompanied by pollinator shifts (e.g. Ellis and Johnson (2009)) and empirical pollination studies are, therefore, required to evaluate predictions.

The flora of the Cape Floristic Region is characterised by a high incidence of specialised pollination systems (Johnson and Steiner 2003; Johnson 2010) and strong predictability of pollination systems based on floral syndromes (Vogel 2012; Johnson and Wester 2017). The monophyletic “Cape clade” of the genus *Erica* is characterised by tremendous floral diversity (Baker and Oliver 1967; Schumann and Kirsten 1992; Vogel 2012). Rebelo et al. (1985) used floral traits to categorise flowers of 426 *Erica* species as consistent with four broadly defined pollination syndromes, distinguishing anemophilous, ornithophilous and entomophilous syndromes and coining the term “rhinomyiophily” to identify the suite of traits indicative of pollination by long-proboscid flies (Rebelo et al. 1985). Although some empirical studies have shown that the syndrome-based categorisations by Rebelo et al. (1985) are not consistently reliable (summarised in Van der Niet (2021)), predictions of pollination by long-proboscid flies (LPF) have been confirmed for several *Erica* species with a characteristic suite of traits including unscented, brightly coloured tubular flowers with a narrow orifice (Lombardi et al. 2021; Newman and Johnson 2021; Pauw 2022; McCarren et al. 2023).

The LPF syndrome of *Erica* flowers is consistent with that of LPF-pollinated species from other plant families (Goldblatt and Manning 2000). Further, narrowly tubular rhinomyiophilous *Erica* flowers restrict access to floral nectar for most other potential pollinators. It is, therefore, likely that predictions based on the LPF syndrome are reliable in *Erica* and that LPF syndrome traits in species which are closely related to species with different floral traits indicate a pollinator shift. *Ericacylindrica*, with narrow floral tubes and upward-facing, pale-coloured and strongly scented flowers, was recently discovered to be pollinated by hawkmoths, a pollination system that is hitherto unique in *Erica* and rare in the CFR (Van der Niet and Cozien 2022). The hawkmoth-pollinated *E.cylindrica* is part of a small clade of species that are all characterised by tubular, upward-facing flowers (Baker and Oliver 1967; Schumann and Kirsten 1992; Pirie et al. 2016); within this clade, *Ericainfundibuliformis* is closely related to *E.cylindrica* (Pirie et al. 2016). Floral traits of the two species show strong similarities, with two key exceptions in colour and scent: flowers of *E.infundibuliformis* are bright pink and not reported to be scented. The combination of apparently unscented, brightly coloured upward-facing flowers and a narrow flower opening conforms to the rhinomyiophilous pollination syndrome and is considered indicative of pollination by LPF in *Erica* (Rebelo et al. 1985). Within LPF-pollinated ericas, flower orientation may also be an important trait for distinguishing pollination by two main groups of LPF pollinators, tabanid and nemestrinid flies. Since tabanid flies are unlikely to feed on upward-facing flowers, plants with such flower orientation are likely pollinated by nemestrinid flies (McCarren et al. 2022; McCarren et al. 2023). Thus, based on differences in floral syndromes, *E.infundibuliformis* was predicted to be pollinated by long-proboscid nemestrinid flies in contrast to pollination by hawkmoths in *E.cylindrica*.

The aim of this study was threefold: firstly, to quantify floral traits to objectively characterise differences between *E.cylindrica* and *E.infundibuliformis*; secondly, to verify predictions of LPF pollination in *E.infundibuliformis* with empirical observations and, finally, to use these combined data to test whether a shift in colour and scent mediates a shift between hawkmoth and nemestrinid fly pollination between the two species.

## ﻿Methods

### ﻿Study species and field sites

**Figure 1.**
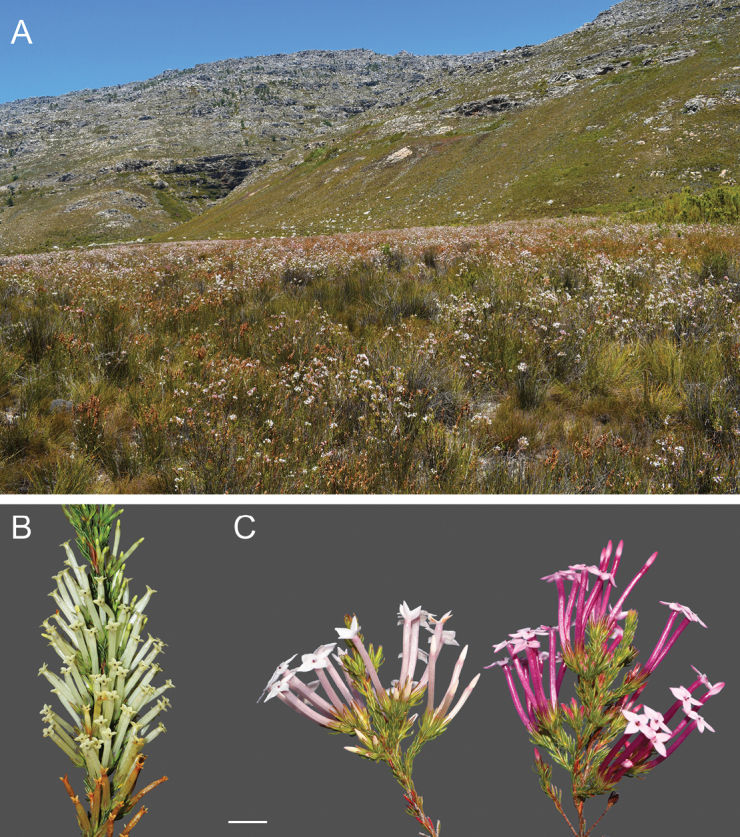
Habitat and flower morphology of the study species. Habitat of *Ericainfundibuliformis* at Agtertafelberg; the white flowers of thousands of *E.infundibuliformis* plants dominate the fynbos of the sandy flats in the foreground (**A**). Inflorescence of *E.cylindrica* from the Voëlvleiberge (**B**). Inflorescences of *E.infundibuliformis* from Agtertafelberg, showing intraspecific flower colour variation (**C**). Scale bar: 10 mm (**B, C**).

*Ericainfundibuliformis* Andr. is distributed along the mountains of the south-western part of the Cape Floristic Region of South Africa (Baker and Oliver 1967). The species grows in fynbos vegetation in damp sandy areas, often in highly localised patches where it may be the dominant plant in the community (Fig. 1A). Fieldwork for this study was carried out at two sites in the Stettynsberg (33.866146°S, 19.325076°E; Stettynsberg hereafter) and Agtertafelberg Mountains (33.800156°S, 19.171326°E; Agtertafelberg hereafter), respectively. Both these sites are in relatively inaccessible parts of the Cape Fold Mountains, which limited opportunities for extensive fieldwork. Plants at the Stettynsberg site had just passed peak flowering on 28–29 November 2023, during which fieldwork was carried out in a patch of several hundreds of plants in a ca. 100 m × 50 m area. Fieldwork at Agtertafelberg was carried out over two consecutive summer seasons: 30 December 2022-1 January 2023, 27–29 December 2023 and 20 January 2024, when the Agtertafelberg population, consisting of many thousands of plants that dominate the vegetation in an area of ca. 500 m × 250 m (Fig. 1A), was in peak flower. All measurements and observations described below mirrored those done for *E.cylindrica* (Van der Niet and Cozien 2022) and were, with the exception of the nectar measurements that were only done at Agtertafelberg, repeated at both study sites.

### ﻿Flower measurements

The length of the corolla tube and angle of flower orientation were measured at both sites. Corolla tube length, the distance from the base of the sepals to the corolla aperture, was measured to the nearest 0.1 mm using digital calipers for three randomly selected flowers per plant on 20 plants per site. Tube length was compared between the populations and with published data for *E.cylindrica* (Van der Niet and Cozien 2022).

The orientation of flowers can be distinguished as upward-facing (ranging from an angle of 30° to vertically upward-facing), horizontally-facing (ranging from an angle of -30° to 30°) and downward-facing (ranging from vertically downward-facing to -30°) (cf. Van der Niet and Cozien 2022). For 30 plants at Stettynsberg and 34 plants at Agtertafelberg, flowers of a randomly selected inflorescence were categorised according to these three classes, based on the methods described in Van der Niet and Cozien (2022) and measurements were compared with those of *E.cylindrica* (Van der Niet and Cozien 2022). Measurements found that almost no flowers face downwards in either of the study species; consequently, there were only two orientation categories and comparisons were, thus, based on the proportion of upward-facing flowers out of the total number of flowers.

### ﻿Spectrophotometry

To quantify flower colour, spectral reflectance was recorded using an Ocean Optics S2000 spectrophotometer, coupled with a DT-mini deuterium-tungsten halogen light source and a fibre optic reflectance probe (QR-400 UVVIS, 400 lm) (Ocean Optics, Inc., Dunedin, Fla.). Reflectance was measured for one flower from each of twenty and twelve different plants at Agtertafelberg and Stettynsberg, respectively. Following the methods used for *E.cylindrica* (Van der Niet and Cozien 2022), two measurements were taken from each *E.infundibuliformis* flower: firstly, from the upper surface of the petal lobe, which is the surface that faces a visitor as it approaches a flower from above or feeds legitimately on nectar and secondly, reflectance of the external surface of the corolla tube was also measured as this may form part of the floral display when flowers are viewed by a visitor approaching from any other angle or by visitors feeding by robbing through punctures in the side of the floral tube. Spectra were imported into R (R Core Team 2021), averaged at 1 nm intervals between 300 and 700 nm and smoothed with a smoothing span of 0.3 nm using the package *pavo* (Maia et al. 2019) to reduce noise for visualisation and for plotting.

To compare spectra from the perspective of LPF, including those of *E.cylindrica* as presented in Van der Niet and Cozien (2022), spectra were also plotted in the categorical colour space developed by Troje (1993). In this colour space, spectra that fall within the same quadrant are considered indistinguishable to flies, whereas spectra located in different quadrants are considered discriminable (Troje 1993). This model is based on spectral sensitivities and experimentally determined discrimination capabilities of *Lucilia* blowflies (Troje 1993), but has also been utilised in studies of pollinator perception of flower colours involving nemestrinid flies (e.g. Whitehead et al. (2018)).

### ﻿Scent sampling

To quantify floral scent emission and characterise the scent bouquet of *E.infundibuliformis*, the headspace of flowering branches was sampled and analysed using gas chromatography coupled with mass spectrometry (GC-MS). Headspace sampling in the field was done according to the protocol described in Van der Niet and Cozien (2022). To quantify differences in per-flower emission rates during day and evening, sampling was repeated twice using the same plants. At Stettynsberg, three plants were sampled from 15:15 h until 16:00 h (daytime) and again from 19:30 h until 20:30 h (evening) on 28 November 2023. These samples were also analysed for scent composition. At Agtertafelberg, four plants were sampled in the evening of 31 December 2022. These samples could not be used to calculate emission rates because the number of flowers was not recorded. For quantifying emission rates at Agtertafelberg, an additional four plants were sampled from 10:50 h until 11:35 h (daytime) and again from 19:55 h until 20:45 h (evening) on 28 December 2023. As these samples contained a large number of minor compounds (n = 62) that could not be identified (making up on average less than 1.5% of the entire blend), composition of these samples is not reported because it was deemed that presenting such a large number of unknown minor compounds would not add any information that can be used for downstream analyses. In all sampling sessions, air from an empty bag was also sampled to control for any compounds present in the ambient air. Samples were stored at -20 °C until further analysis.

Samples were run on the same Varian CP-3800 gas chromatograph with a 30 mm × 0.25 mm internal diameter (film thickness 0.25 μm) Alltech EC-WAX column, coupled to a Bruker 300-MS quadrupole mass spectrometer as was used to analyse the evening samples of *E.cylindrica* (Van der Niet and Cozien 2022), under an identical temperature programme. Compound identification followed the same procedure as described in Van der Niet and Cozien (2022), although a more recent version of the NIST Mass Spectral Search Program (version 2.4, 2020) was used. Mass spectra of compounds that could not be identified and that were found across all samples in a batch or across multiple samples in multiple batches, are presented in Suppl. material 1, arranged by ascending Kovats Retention Index.

### ﻿Nectar

Standing crop nectar volume and sugar concentration were measured from flowers from Agtertafelberg in 2024. Twenty-four inflorescences, each one sampled from a different plant, were collected at 10:00 h in the morning, kept cool with stems in water and measured in the laboratory at 18:00 h on the same day. Nectar volume was measured from one randomly selected flower per inflorescence by cutting the base of the flower and gently squeezing the liquid into graduated 5 μl glass micro-capillary tubes. Sugar concentration from flowers that produced more than 0.1 μl of nectar was measured as % Brix by spotting the nectar on to a hand-held Bellingham & Stanley pocket sugar refractometer. Nectar volume and sugar concentration of *E.cylindrica* as reported in Van der Niet and Cozien (2022) are presented for comparison.

### ﻿Pollinator observations

Pollinator observations were carried out over six days and three nights, for a total of 31 observer hours, of which one third were during the evening, over the 2021–2022 and 2022–2023 flowering seasons at Agtertafelberg. Observations at Stettynsberg were limited to a single day and evening (total five and a half hours, all with two observers) in the 2023 flowering season. At both sites, observations included morning, afternoon and evening hours from 07:30 h until 21:00 h, to increase the likelihood of observing both diurnal and nocturnal visitors.

Visitor behaviour was observed and photographed, to distinguish legitimate visits involving insertion of insects’ proboscides into the floral tube, facilitating contact with reproductive parts, from illegitimate robbing visits in which visitors fed on nectar though a hole in the base of the floral tube, without potential for contacting anthers or stigma. Visitors were identified according to their functional pollinator group at the level of insect genera, families or superfamilies. No flower visits by vertebrates were observed. For identification, and to assess potential of different visitors as pollen vectors, 1–10 (median n = 3) representatives of each functional pollinator group were captured with a hand-held sweep net, immediately transferred to Eppendorf tubes and then kept in a freezer until processing. Insect bodies were sampled in the laboratory for pollen grains using a 1 mm^3^ cube of fuschin gel (Beattie 1971), which was subsequently melted on to a microscope slide for pollen counts using a Zeiss Lab.A1 light microscope. *Ericainfundibuliformis* produces pollen in tetrads (unpublished data). Pollen counts distinguished between *Erica* tetrad pollen and other pollen grains. Although several other *Erica* species flowered simultaneously, *E.infundibuliformis* was by far the most common *Erica* species in the community where visitors were caught and, with the exception of a single fly (see Results), all insects were caught while visiting *E.infundibuliformis*. We, therefore, assume that the majority of *Erica* tetrads on caught insects were from *E.infundibuliformis*. Proboscis length of the caught visitors was measured to the nearest 0.1 mm using a pair of digital calipers. Voucher specimens of representative insects were submitted to the KwaZulu-Natal Museum or University of KwaZulu-Natal collections.

### ﻿Nectar robbing and anther ring disruption

Rates of both legitimate visitation and illegitimate (nectar robbing) visitation in an *Erica* population can be quantified indirectly, without the need for direct observations, from physical evidence. Damage to the tissue of floral tubes is indicative of nectar robbing, whereas legitimate visits by pollinators results in disruption of the anthers that are fused in a ring surrounding the style (Baker and Oliver 1967; Geerts and Pauw 2011). For the same flowers as for which corolla tube length was measured, signs of nectar robbing in the form of a pierced corolla tube and pollination in the form of anther ring disruption were evaluated and compared between the two populations.

### ﻿Statistical analyses

Floral phenotypic traits were compared using Generalised Linear Models (GLM). The continuous morphometric traits ‘floral tube length’ and ‘floral scent emission rate’ were both modelled with a gamma distribution and log link function. Comparisons of evening scent emission rates amongst the single *E.cylindrica* population and the two *E.infundibuliformis* populations were analysed with GLM, whereas variation in diurnal and evening scent emission rates between *E.infundibuliformis* plants from Stettynsberg and Agtertafelberg was analysed using Generalised Estimating Equations (GEE) with “plant” as subject variable and “time period” as within-subject variable and an exchangeable correlation matrix, to account for repeated measures of the same plant. We tested for an effect of time period, population and the interaction between these factors. Variation in corolla tube length amongst *E.cylindrica* and the two *E.infundibuliformis* populations was analysed using GEE to account for correlations amongst flowers measured on the same plant individual, with “plant” as the subject variable and “flowers” as within-subject variables and an exchangeable correlation matrix. Flower orientation, as the number of upward-facing flowers out of the number assessed on each inflorescence, was modelled using a binary logistic distribution and logit link function. Variation in scent composition was visualised using non-metric multi-dimensional scaling, based on Bray-Curtis similarity of square-root transformed proportions of compounds amongst samples, including the samples of *E.cylindrica* that were reported in Van der Niet and Cozien (2022). Similarity in scent composition between *E.cylindrica* and *E.infundibuliformis* was statistically compared between species using an ANalysis Of SIMilarity (ANOSIM), whereas the compounds that contribute most to dissimilarity between the species were identified using a Similarity Percentage (SIMPER) analysis. These analyses were conducted as described in Van der Niet and Cozien (2022), using PAST 4.03 (Hammer et al. 2001). Nectar volume and sugar concentration of *E.infundibuliformis* flowers were compared to those of *E.cylindrica* (Van der Niet and Cozien 2022) using a Mann-Whitney U-test singular in PAST 4.03 (Hammer et al. 2001), due to the small and highly unequal sample sizes and nature of the data. Nectar robbing and anther ring disturbance were compared between populations using a GLM with number of affected flowers out the number assessed (almost always three) modelled with a binary logistic distribution and logit link function. In case a log or logit link function was used, means and standard errors were back-transformed to the original scale for graphing purposes, resulting in asymmetrical error bars. All statistical analyses were carried out in in SPSS v. 29 (IBM, Corp.), unless mentioned otherwise.

## ﻿Results

**Figure 2.**
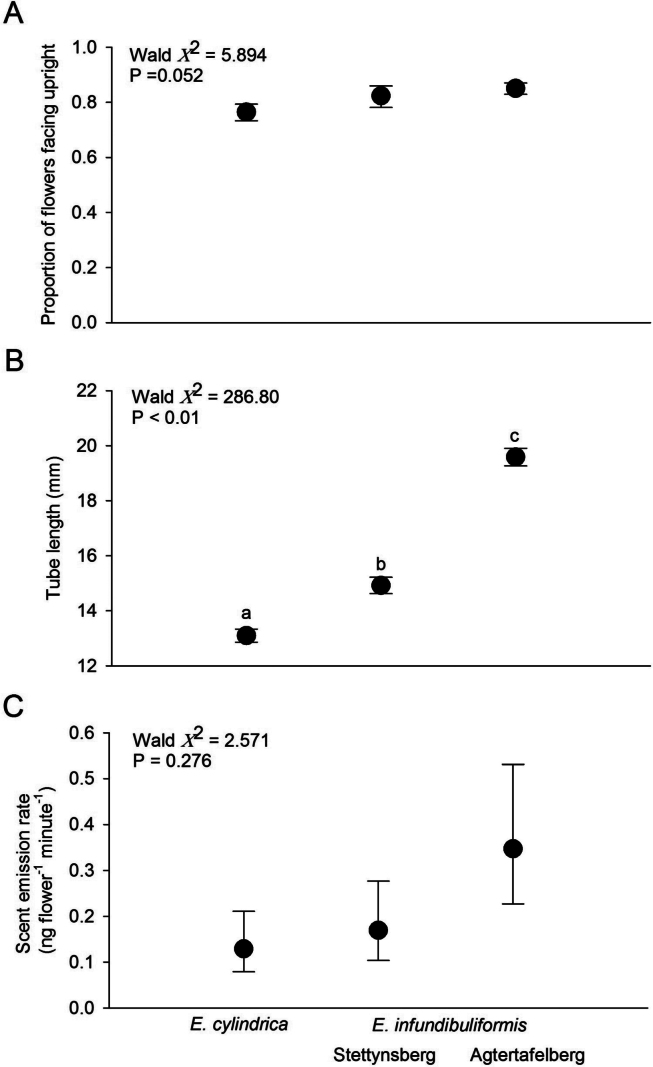
Comparisons of floral and inflorescence characteristics between *Ericacylindrica* and populations of *E.infundibuliformis*: flower orientation (**A**), corolla tube length (**B**) and evening scent emission rates (**C**). Letters indicate significant pairwise comparisons at P < 0.05 (**B**).

Corolla length differed significantly amongst all three populations (Figs 1, 2) and was shortest in *E.cylindrica* and longest in *E.infundibuliformis* from Agtertafelberg. In both species, more than 75% of flowers of an inflorescence faced upright and this proportion did not differ amongst the populations (Fig. 2).

**Figure 3.**
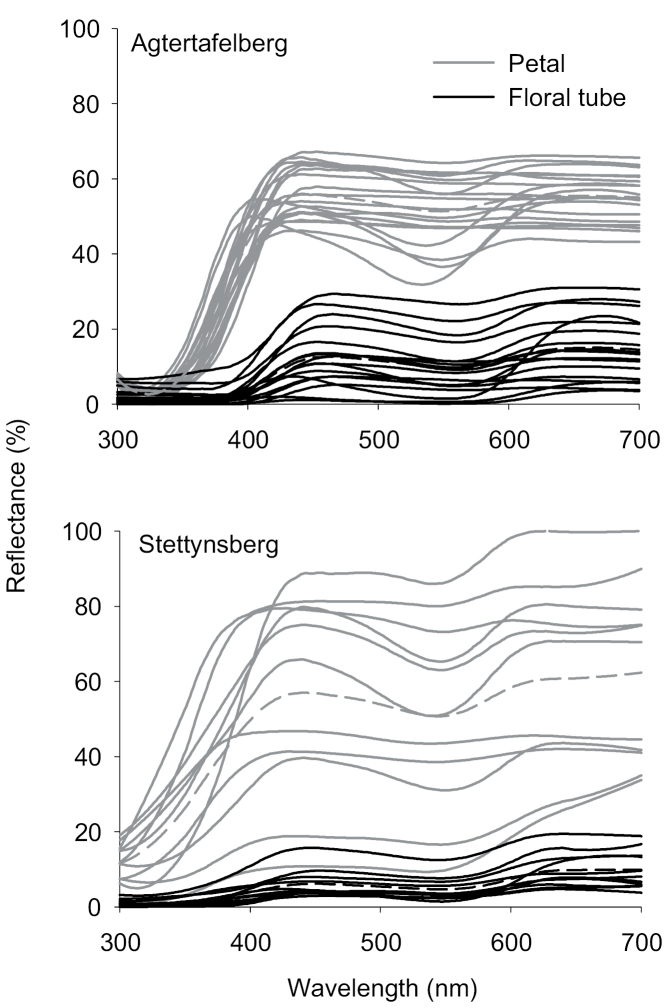
Reflectance of floral petal surfaces (grey) and outer surfaces of corolla tubes (black) of *Ericainfundibuliformis* from Agtertafelberg and Stettynsberg. Solid lines represent reflectance measurements recorded from individual flowers, dashed lines show means for all spectra recorded for the respective floral parts in each population.

Floral spectral reflectance patterns were largely similar for flowers of both populations of *E.infundibuliformis*, despite some variation in brightness (Fig. 3). Petals of flowers from both populations of *E.infundibuliformis* had greater overall brightness than flowers of *E.cylindrica* and also were consistently characterised by a steep increase to maximum reflectance in the UV region, around 350 nm (Fig. 3), which was almost absent in flowers of *E.cylindrica*. Overall brightness varied somewhat amongst flowers within both *E.infundibuliformis* populations, but was usually consistently close to maximum between 350 and 700 nm, with an average of approximately 50% for petals and 10% for floral tube surfaces (Fig. 3), which is approximately double that observed for flowers of *E.cylindrica*. The variation in flower colour within *E.infundibuliformis* populations was mostly in the degree of reflectance between 500 and 600 nm (Figs 1, 3).

**Figure 4.**
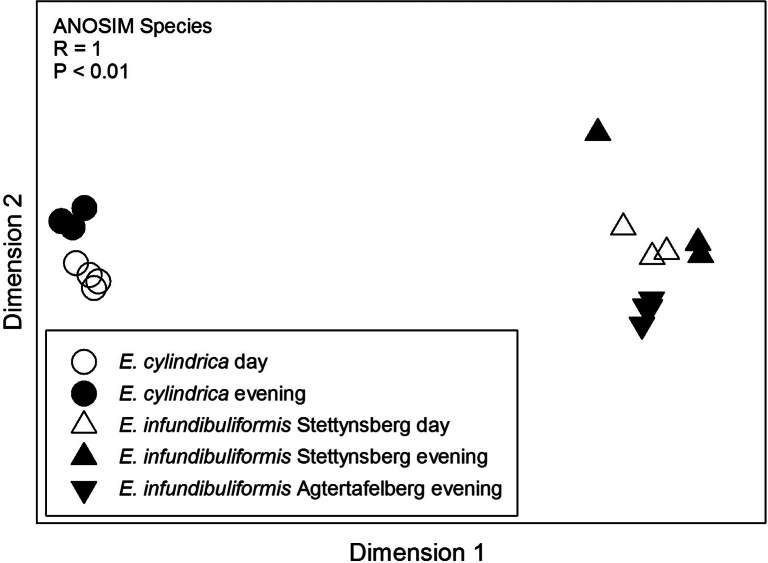
Non-metric multidimensional scaling of daytime and evening scent bouquets of *Ericacylindrica* and *E.infundibuliformis* populations.

**Figure 5.**
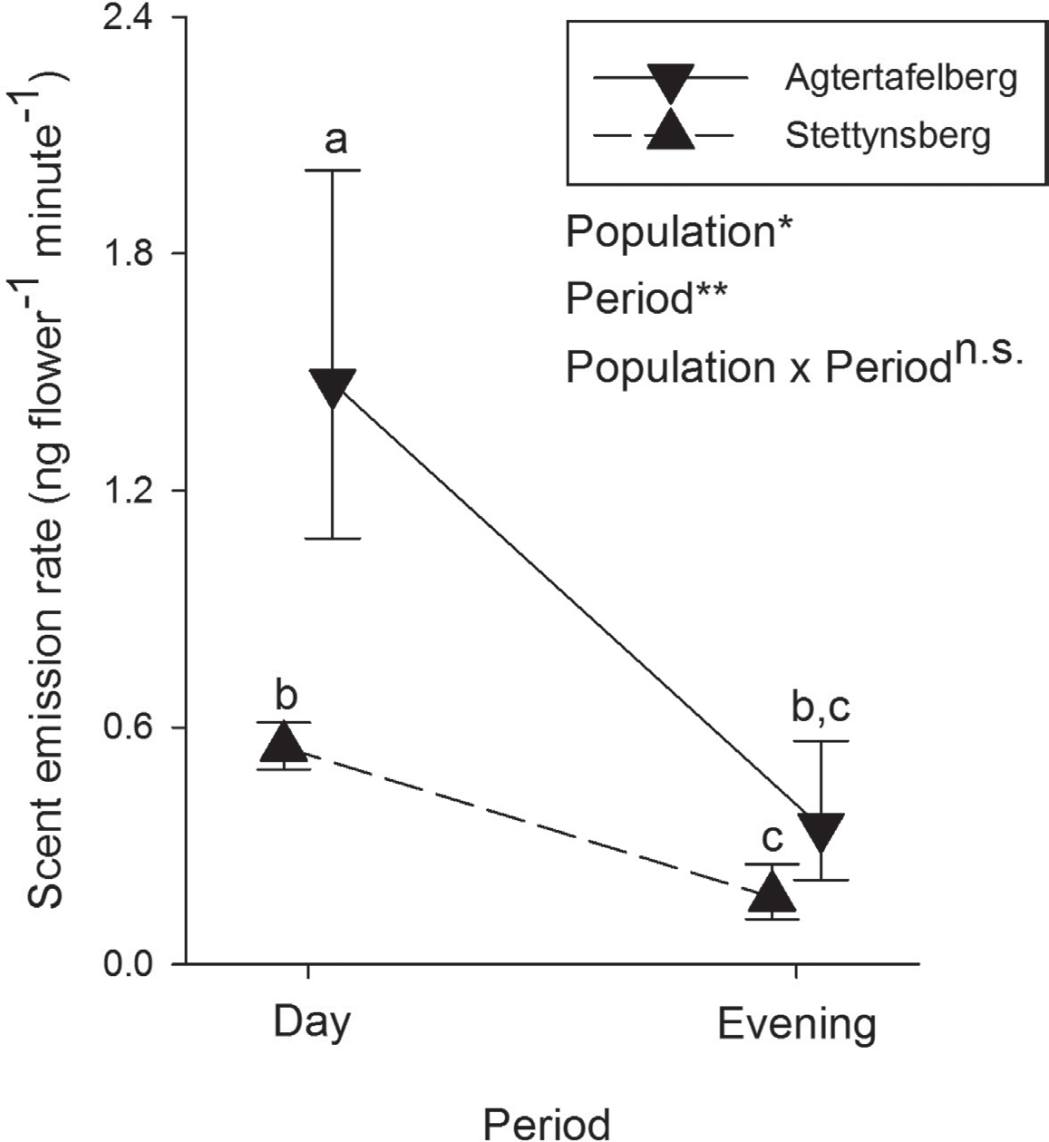
Comparison of daytime and evening scent emission rates in the two *Ericainfundibuliformis* study populations. Different letters indicate significant pairwise differences in emission rates at P < 0.05. Asterisks refer to levels of significance: ** P < 0.01, * P < 0.05.

A total of 82 compounds were detected in the scent samples of *E.infundibuliformis* (Table 1). Just over half of these (n = 42) could not be identified, but these unknown compounds on average only made up between 0.49% and 2.87% of the entire blend. The largest number of identified compounds were monoterpenes and these were also the most dominant in terms of scent emission, with (E)-ocimene being the most important compound at both sites and during both day and evening (Table 1). Of the 48 compounds produced by *E.cylindrica*, only ten were in common with *E.infundibuliformis* and the bouquets of these two species were consequently significantly different in composition (Fig. 4). The main difference between the species involved the unique presence of a large number of dominant aromatic compounds in *E.cylindrica*, contrasting with the large number of dominant monoterpenes in *E.infundibuliformis* (Table 2). Evening emission rates did not differ significantly amongst the populations (Fig. 2), whereas there was an effect of both population and period (but no significant interaction) in the comparison of daytime and evening scent emission rates of the two *E.infundibuliformis* populations: emission rates were higher at Agtertafelberg and during daytime (Fig. 5).

**Table 1.** Percentage (mean ± SD) of each compound as part of the headspace of *Ericainfundibuliformis*. Compounds are grouped by major compound class (cf. Knudsen and Tollsten 1993) and sorted within class according to the Kovats Retention Index (KRI). CAS number is provided for compounds that could be identified. In case a compound could be identified without information of which the particular stereoisomer was present, no CAS number is provided. Compound names in cells with light grey shading were also found in *E.cylindrica*. Sample sizes of percentages are only given in case a compound was not found in all samples within a particular batch. Mass spectra of compounds for which the KRI is marked with an asterisk are provided in Suppl. material 1.

Compound name	KRI	CAS number	Stetteynsberg day (n = 3)	Stetteynsberg evening (n = 3)	Agtertafelberg evening (n = 4)
**Aliphatics**					
***Aliphaticalcohols***					
(E)-Hex-3-en-1-ol	1364	928-97-2	1.57 ± 2.50	0.02 ± 0.02 (2)	1.46 ± 0.63
Oct-1-en-3-ol	1426	3391-86-4	0.02 ± 0.02	0.02 ± 0.02 (2)	0.67 ± 0.30
4-Hexen-3-ol	1754	4798-58-7	0.03 ± 0.02 (2)	0.04 ± 0.03 (2)	0.18 ± 0.13
***Aliphaticaldehydes***					
(E)-Hex-2-enal	1213	6728-26-3			0.09 ± 0.10
(E)-4-Oxohex-2-enal	1568	20697-55-6	0.51 ± 0.83		0.65 ± 0.50
***Aliphaticalkanes***					
Tetradecane	1400	629-59-4			0.06 ± 0.13 (1)
Pentadecane	1500	629-62-9			0.12 ± 0.24 (1)
Hexadecane	1600	544-76-3			0.14 ± 0.29 (1)
Heptadecane	1700	629-78-7			0.05 ± 0.11 (1)
Octadecane	1800	593-45-3			0.03 ± 0.05 (2)
***Aliphaticesters***					
(E)-Hex-4-en-1-yl acetate	1302	72237-36-6	1.05 ± 0.77		0.95 ± 0.69
(E)-3-Hexen-1-yl butyrate	1445	53398-84-8	0.40 ± 0.58		0.39 ± 0.23
(Z)-3-hexenyl 2-methylbutyrate	1460	53398-85-9	0.03 ± 0.04		0.03 ± 0.01
(Z)-3-Hexenyl hexanoate	1638	31501-11-8	0.03 ± 0.02		
**Benzenoids**					
Benzaldehyde	1503	100-52-7	0.11 ± 0.04	3.20 ± 5.47	0.18 ± 0.09
Phenylethyl alcohol	1881	60-12-8	0.02 ± 0.00	0.19 ± 0.31	0.03 ± 0.01
**Isoprenoids**					
***Irregularterpene***					
6-Methyl-5-hepten-2-one	1322	110-93-0	0.10 ± 0.05	0.04 ± 0.07 (1)	0.96 ± 0.67
***Monoterpenes***					
β-Myrcene	1163	123-35-3	1.13 ± 0.44	0.65 ± 0.57 (2)	0.95 ± 0.36
(Z)-Ocimene	1231	3338-55-4	5.36 ± 1.59	1.20 ± 1.05 (2)	3.79 ± 0.40
(E)-Ocimene	1251	3779-61-1	80.0 ± 5.02	89.1 ± 3.15	82.9 ± 2.06
2,6-Dimethylocta-2,4,6-triene stereoisomer 1	1367		0.39 ± 0.40		0.15 ± 0.07
2,6-Dimethylocta-2,4,6-triene stereoisomer 2	1384		0.47 ± 0.81 (1)		
2,6-Dimethyl-1,3,5,7-octatetraene stereoisomer 1	1423		0.05 ± 0.06	0.09 ± 0.10 (2)	0.28 ± 0.18
2,6-Dimethyl-1,3,5,7-octatetraene stereoisomer 2	1435		0.35 ± 0.28	0.34 ± 0.38 (2)	1.58 ± 0.48
(Z)-Furan linalool oxide stereoisomer	1453		0.03 ± 0.01	0.01 ± 0.02 (1)	
Myroxide stereoisomer	1469		0.06 ± 0.05 (2)	0.04 ± 0.03 (2)	0.10 ± 0.04
Linalool	1520	78-70-6	6.49 ± 5.66	4.01 ± 2.81	
Cinerone stereoisomer	1542*		0.04 ± 0.03 (2)	tr (1)	0.12 ± 0.09
Pinocarvone	1561	30460-92-5	0.03 ± 0.03 (2)	0.07 ± 0.10 (2)	
α-Terpineol	1672	98-55-5	0.02 ± 0.01		
Pinocarveol	1685	5947-36-4	0.06 ± 0.01	0.01 ± 0.01 (1)	0.07 ± 0.04
p-Mentha-1,5-dien-8-ol	1695	1686-20-0	tr (2)	0.01 ± 0.01 (2)	0.02 ± 0.02 (3)
2,6-dimethylocta-3,5,7-trien-2-ol stereoisomer 1	1770		tr (2)	0.03 ± 0.02 (2)	0.08 ± 0.07 (3)
2,6-dimethylocta-3,5,7-trien-2-ol stereoisomer 2	1787		0.11 ± 0.10 (2)	0.22 ± 0.19 (2)	0.59 ± 0.28
2,6-Dimethyl-3,7-octadiene-2,6-diol	1900*	13741-21-4	0.02 ± 0.01 (2)		
**Miscellaneous compounds**					
3-Methyl-2-(2-methyl-2-butenyl)-furan	1389	15186-51-3	0.10 ± 0.06	0.03 ± 0.03 (2)	0.11 ± 0.04
5,5-dimethyl-2(rH)-furanone	1583	20019-64-1	0.03 ± 0.02 (2)	0.02 ± 0.02 (2)	0.01 ± 0.03 (1)
5-Methyl-5-vinyldihydrofuran-2(3H)-one	1648	1073-11-6	0.01 ± 0.01 (2)	0.01 ± 0.01 (1)	
**Nitrogen-containing compounds**					
3-Methylpyrazole	1654	1453-58-3	tr (2)	0.02 ± 0.01 (2)	0.16 ± 0.08
Benzyl isocyanide	1657	10340-91-7	0.08 ± 0.03	0.02 ± 0.02 (2)	0.01 ± 0.00
**Unknown compounds**					
m/z: 53,81,82,54,50,55	1121*		0.46 ± 0.17	0.18 ± 0.31 (1)	0.32 ± 0.16
m/z: 91,96,119,67,95,41	1358*		0.07 ± 0.02	0.06 ± 0.05 (2)	0.36 ± 0.20
m/z: 73,56,59,86,72,55	1464*		0.05 ± 0.04	0.03 ± 0.04 (2)	
m/z: 91,107,43,92,65,79	1490*		0.03 ± 0.03 (2)	0.01 ± 0.01 (2)	0.13 ± 0.11
m/z: 55,43,32,83,41,42	1501				0.02 ± 0.02 (3)
m/z: 95,93,123,67,91,81	1511*		0.03 ± 0.02 (2)	0.01 ± 0.01 (1)	0.06 ± 0.01
m/z: 57,85,86,43,55,72	1524*				0.28 ± 0.22
m/z: 95,93,79,41,55,69	1525				0.06 ± 0.00
m/z: 82,83,55,41,53,39	1546				0.01 ± 0.01 (2)
m/z: 43,71,57,70,41,55	1551				0.01 ± 0.02 (1)
m/z: 108,82,79,42,80,81	1650*				0.03 ± 0.01
m/z: 57,71,43,41,55,85	1666				0.01 ± 0.03 (1)
m/z: 60,91,73,107,79,150	1700*		0.03 ± 0.00	0.01 ± 0.01 (2)	0.09 ± 0.05
m/z: 83,55,84,57,82,112	1730		0.05 ± 0.10 (1)		
m/z: 57,43,71,55,84,41	1742				0.01 ± 0.03 (1)
m/z: 82,67,71,43,81,79	1771*		0.04 ± 0.01	tr (1)	0.02 ± 0.05 (1)
m/z: 95,54,43,59,81,67	1844*		0.01 ± 0.02 (1)	tr (2)	0.04 ± 0.02
m/z: 95,43,55,59,81,67	1848*		tr (1)	tr (2)	0.02 ± 0.00
m/z: 43,95,110,59,81,71	1890*				0.04 ± 0.01
m/z: 57, 85, 43, 41, 55, 39	1925*		0.18 ± 0.20	0.01 ± 0.02 (2)	0.51 ± 0.46 (3)
m/z: 153,109,83,69,43,32	1940*				tr (2)
m/z: 71,43,41,39,53,69	1941*		tr (2)	tr (2)	0.01 ± 0.01 (3)
m/z: 59,71,43,53,55,113	1946*			0.01 ± 0.00 (2)	0.03 ± 0.02
m/z: 59,42,71,55,41,113	1951*		tr (1)		0.06 ± 0.05 (3)
m/z: 97,72,43,68,95,79	1964*		0.07 ± 0.07 (2)	0.02 ± 0.02 (2)	0.11 ± 0.04
m/z: 43,125,83,107,81,55	1971*				0.01 ± 0.02 (3)
m/z: 43,57,69,41,55,91	1972				tr (1)
m/z: 97,67,41,72,68,43	1987*		0.01 ± 0.01 (2)	0.01 ± 0.01 (2)	0.06 ± 0.03
m/z: 59,43,71,113,73,83	1993*				0.04 ± 0.02
m/z: 71,59,43,85,113,73	2004*				0.02 ± 0.01
m/z: 79,91,150,39,107,32	2030*		0.02 ± 0.00	0.01 ± 0.01 (2)	0.04 ± 0.01
m/z: 58,43,71,59,55,445	2104				tr (1)
m/z: 43,111,32,41,91,93	2124*		tr (1)	0.01 ± 0.00 (2)	0.16 ± 0.13
m/z: 79,108,77,39,80,82	2127*		tr (1)	tr (2)	0.02 ± 0.00
m/z: 43,95,59,41,55,79	2134*		tr (1)	tr (2)	tr (1)
m/z: 121,149,138,194,93,65	2137				tr (1)
m/z: 43,95,32,55,97,59	2141*		tr (1)	0.01 ± 0.01 (2)	
m/z: 95,43,97,41,83,59	2150*		tr (1)	0.02 ± 0.02 (2)	0.02 ± 0.01
m/z: 109,79,81,152,67,121	2154*		tr (1)	0.01 ± 0.01 (2)	0.03 ± 0.02
m/z: 74,87,43,41,55,75	2196				tr (1)
m/z: 88,43,100,41,54,30	2236				tr (1)
m/z: 69,93,41,81,79,91	2257				0.01 ± 0.01 (2)
**Aliphaticalcohols**			**1.63 ± 2.50**	**0.09 ± 0.08**	**2.32 ± 0.96**
**Aliphaticaldehydes**			**0.51 ± 0.83**	**0**	**0.75 ± 0.59**
**Aliphaticalkanes**			**0**	**0**	**0.42 ± 0.83**
**Aliphaticesters**			**1.53 ± 1.41**	**0**	**1.39 ± 0.91**
**Benzenoid compounds**			**0.14 ± 0.04**	**3.40 ± 5.79**	**0.22 ± 0.10**
**Irregularterpene**			**0.10 ± 0.05**	**0.04 ± 0.07**	**0.96 ± 0.67**
**Monoterpenes**			**93.55 ± 5.46**	**95.19 ± 4.57**	**89.77 ± 1.97**
**Miscellaneous compounds**			**0.15 ± 0.03**	**0.06 ± 0.06**	**0.13 ± 0.05**
**nitrogen-containing compounds**			**0.08 ± 0.04**	**0.04 ± 0.03**	**0.17 ± 0.07**
**Unknown compounds**			**1.13 ± 0.33**	**0.49 ± 0.50**	**2.87 ± 0.75**

**Table 2.** Results from the Similarity Percentage (SIMPER) analysis comparing the scent bouquets of *Ericacylindrica* and *E.infundibuliformis*. Listed are the 20 compounds that contribute the most to dissimilarity, which together contribute almost 70% of the entire dissimilarity, arranged in descending order of contribution.

Compound name	Compound class	Cumulative contribution to dissimilarity (%)	Mean proportion *E.cylindrica*	Mean proportion *E.infundibuliformis*
(E)-Ocimene	Monoterpene	15.40	0.041	0.916
Benzyl alcohol	Benzenoid compound	24.99	0.534	0
Benzyl acetate	Benzenoid compound	34.16	0.517	0
Benzaldehyde	Benzenoid compound	39.09	0.347	0.062
Eugenol	Benzenoid compound	42.26	0.181	0
(Z)-Hex-3-en-1-ol	Aliphatic alcohol	45.42	0.192	0
(Z)-Ocimene	Monoterpene	48.37	0	0.174
(E)-Hex-4-en-1-yl acetate	Aliphatic ester	50.85	0.181	0.067
Hexyl acetate	Aliphatic ester	53.23	0.141	0
Linalool	Monoterpene	55.59	0	0.128
Methyleugenol	Benzenoid compound	57.28	0.097	0
β-Myrcene	Monoterpene	58.82	0	0.09
Hexan-1-ol	Aliphatic alcohol	60.27	0.087	0
Octyl acetate	Aliphatic ester	61.67	0.081	0
(E)-5-Decen-1-ol, acetate,	Aliphatic ester	63.06	0.086	0
2,6-Dimethyl-1,3,5,7-octatetraene	Monoterpene	64.42	0	0.081
(Z)-Methyl isoeugenol	Benzenoid compound	65.65	0.071	0
Pentyl acetate	Aliphatic ester	66.85	0.074	0
(E)-Hex-3-en-1-ol	Aliphatic alcohol	68.02	0.021	0.080
(E)-4-Oxohex-2-enal	Aliphatic aldehyde	68.95	0.035	0.046

Flowers of *E.infundibuliformis* produced a mean ± SD of 0.49 ± 0.42 μl of nectar (n = 24 flowers), with a sugar concentration of 35.8 ± 10.9% (n = 19 flowers), whereas flowers of *E.cylindrica* produced a mean ± SD of 0.19 ± 0.17 μl of nectar (n = 10 flowers), with a sugar concentration of 24.1 ± 5.3% (n = 6 flowers). Both nectar volume and sugar concentration were higher for *E.infundibuliformis* compared to *E.cylindrica* (nectar volume: z = 2.052, P < 0.05; sugar concentration: z = 2.39, P < 0.05).

**Figure 6.**
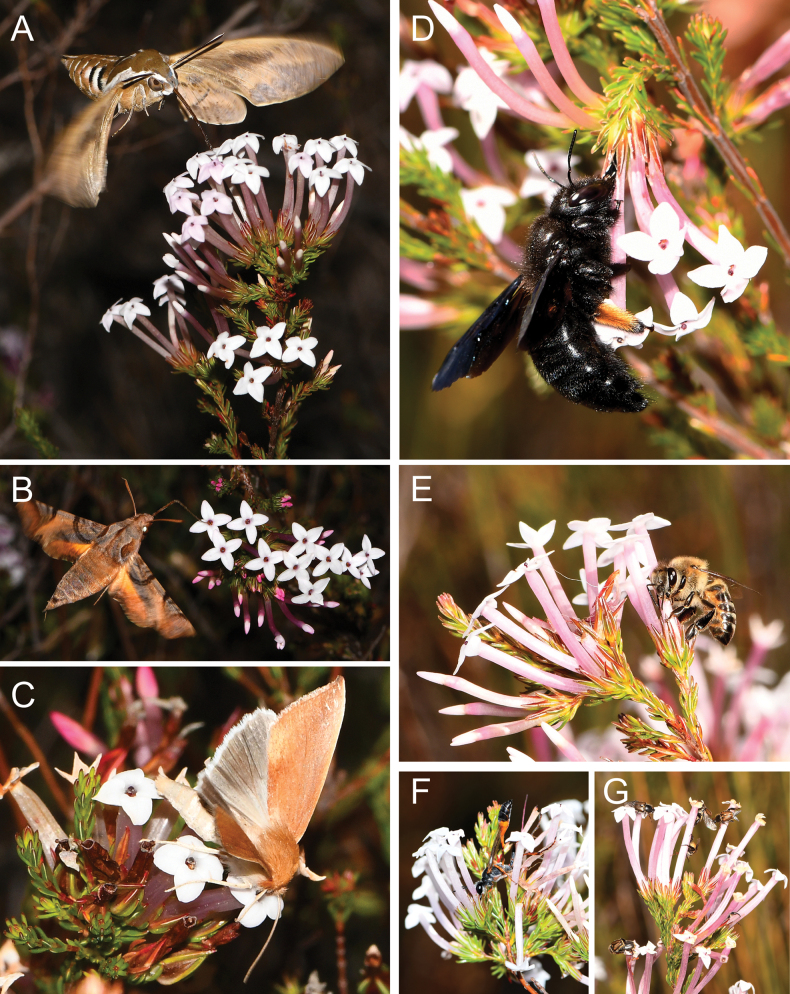
Interactions between *Ericainfundibuliformis* and flower visitors. Legitimate visits with potential for effective pollen transfer are shown on the left: *Hippotionosiris* with white pollen grains visible along the proboscis (**A**), *Temnora* sp. (**B**), both at Agtertafelberg; and Geometrid moth (possibly *Acrasia* sp.) visiting a flower at Stettynsberg (**C**). Illegitimate robbing visits, all photographed at Agtertafelberg, are shown on the right: *Xylocopa* sp. (D), *Apismelliferacapensis* (**E**) and a Sphecidae wasp sp. (**F**) feed on nectar through a puncture in the base of the floral tube without contacting reproductive parts of flowers. *Hopliini* sp. visiting flowers, possibly feeding on floral tissue (**G**).

At both study sites, moths were the most frequently observed visitors that fed legitimately from *E.infundibuliformis* flowers (Table 3, Fig. 6). At Agtertafelberg, a total of 12 hawkmoths were observed feeding on flowers of *E.infundibuliformis* over all three evenings of observations. At Stettynsberg, five hawkmoths were observed on a single evening, in addition to 15 settling moths which were observed feeding during daytime and evening hours (Table 3). Both hawkmoths and settling moths carried up to 1000 pollen grains per individual (overall mean ± SD 540.7 ± 362.9 grains, n = 11), of which on average 97% constituted *Erica* tetrads (SD = 7.4%, n = 11); 90% of *Erica* pollen carried by moths was located on moths’ proboscides (mean ± SD 90.1 ± 18.6%, n = 11) (Table 3).

**Table 3.** Flower visitors of *Ericainfundibuliformis* at both study sites, including tongue lengths and pollen loads. Numbers represent mean ± SD (sample size), apart from visitors observed, which are counts.

	Number observed	Tongue length (mm)	Pollen load	Percent *Erica* tetrads in pollen
**Agtertafelberg**				
**Legitimate feeding**				
Hawkmoth spp.	12	20.7 ± 0.92 (3)	259.3 ± 33.9 (3)	88.2 ± 10.2 (3)
**In population**				
*Moegistorhynchus* sp.	1	19.0 (1)	245 (1)	100
**Robbing**				
*Xylocopa* sp.	121	5.94 ± 1.33 (8)	187.9 ± 392.3 (11)	0.44 ± 1.3 (9)
Other bee sp.	3	3.24 (2)	2768.0 ± 3805.7 (3)	1.6 ± 1.2 (3)
Wasp sp.	12	2.01 ± 0.37 (4)	66.5 ± 111.7 (4)	25.0 ± 43.3 (4)
**On plant**				
Monkey beetles	10	0.9 (1)	0	0
**Stettynsberg**				
**Legitimate feeding**				
Hawkmoth spp.	5	19.6 (2)	851.0 ± 67.2 (3)	99.5 ± 0.44 (3)
Settling moth spp.	15	12.3 ± 3.27 (4)	523.4 ± 425.2 (5)	100 ± 0.0 (5)
**In vicinity**				
*Philoliche* sp.	5	25.5 ± 0.41 (3)	1020.3 ± 484.3 (3)	0 (3)

No dipteran visitors to flowers of *E.infundibuliformis* were observed at Stettynsberg, despite the presence of several individuals of *Philoliche* (Tabanidae) which visited Iridaceae (*Tritoniopsiscooperi*, *Geissorhizaconfusa*) and Proteaceae (*Serruria*) species in close proximity to *Erica* at this study site. Pollen on captured individuals of *Philoliche* also did not include *Erica* pollen (Table 3). At Agtertafelberg, one Bombyliid (*Exoprosopa* sp.) was observed foraging on flowers of *E.infundibuliformis* and one nemestrinid, *Moegistorhynchus* sp. nov., was captured in the population and carried approximately 250 grains of exclusively *Erica* pollen.

Floral larceny was observed almost constantly during diurnal observations at Agtertafelberg, but was not observed at Stettynsberg. At Agtertafelberg, 136 incidences of insects feeding through slits in the side of floral tubes, without potential for contact with reproductive parts, were observed, of which 90% were by carpenter bees and the balance by other hymenopterans including wasps and honeybees (Table 3). Monkey beetles were present on flowers, but did not feed on any flower parts (Table 3). Of 18 captured representatives of insects considered to exhibit robbing behaviour, only one individual carried more than ten grains of *Erica* pollen (Table 3). Carpenter bees captured on *E.infundibuliformis* flowers carried an average of 188 pollen grains (SD = 392.38, n = 10), of which less than 1% were *Erica* tetrads (mean ± SD 0.44 ± 1.26%, n = 9).

**Figure 7.**
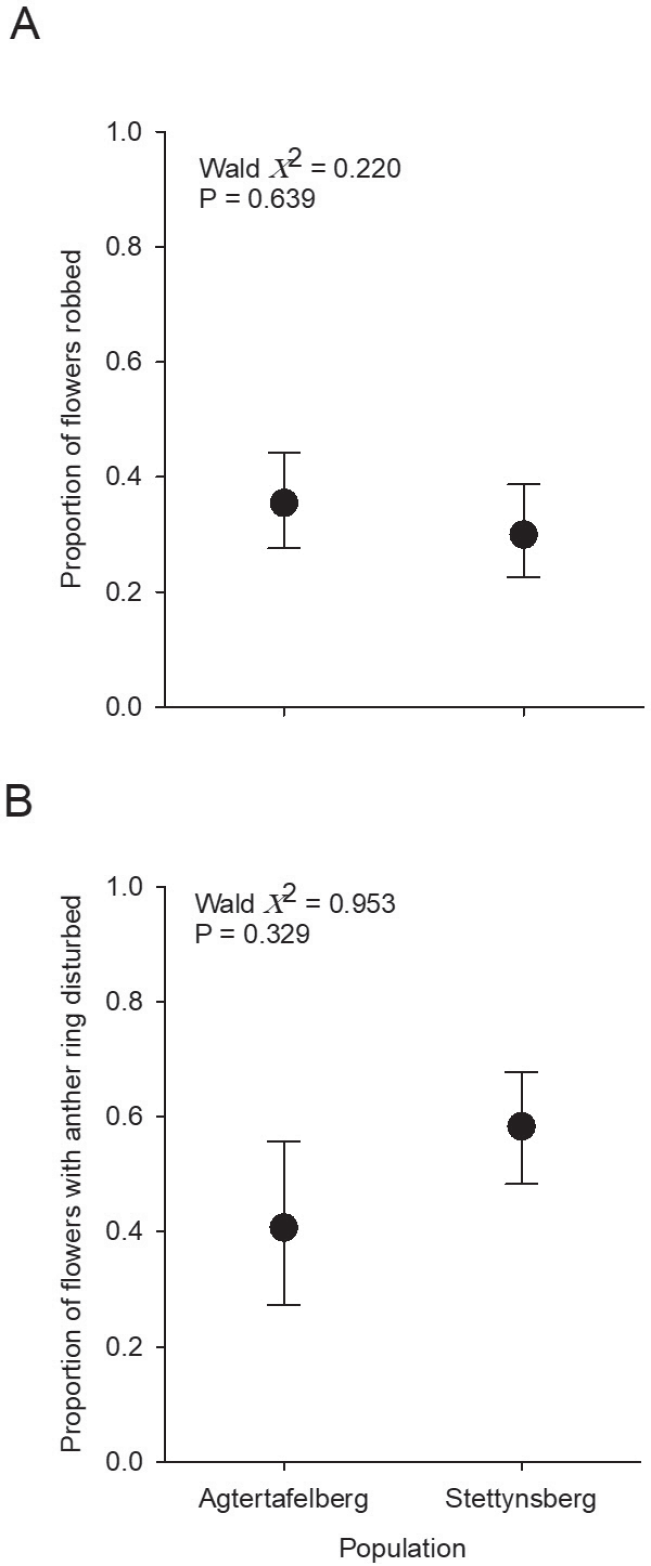
Frequencies of nectar robbing and anther ring disruption (as a proxy for pollination rate) in the two *Ericainfundibuliformis* study populations.

Rates of nectar robbing (as assessed from the proportion of flowers with evidence of puncturing in the tissue of the floral tube) varied between 30% (Stettynsberg) and 35% (Agtertafelberg), whereas anther ring disruption varied between 41% (Agtertafelberg) and 58% (Stettynsberg) of assessed flowers, but neither differed significantly between sites (Fig. 7).

## ﻿Discussion

Results from this study revealed divergence in several floral traits, including scent, colour, corolla tube length and nectar, between *E.cylindrica* and *E.infundibuliformis*. Despite these floral differences, pollinator observations revealed that both species are pollinated by moths, contrary to the idea that the differences in floral traits indicate a difference in pollination system.

Although *E.infundibuliformis* was visited by a large number of insect species during both daytime and the evening, legitimate visits were almost exclusively limited to settling moths and hawkmoths, which carried large amounts of *Erica* tetrads. These observations strongly contradict the expectation that the pollination system of *E.infundibuliformis* differs from that of the hawkmoth-pollinated *E.cylindrica*. Moth visits were observed consistently on all evenings, over multiple years and at two different sites with somewhat different flowering phenology; the two study sites have non-overlapping flowering periods, such that by the time of peak flowering in December-January at Agtertafelberg, flowering at Stettynsberg – which peaks in November – is completely over. Consistent observations of moth pollination, in combination with the fact that pollen loads on moths consisted largely of *Erica* pollen, therefore suggests an established plant-pollinator interaction, rather than opportunistic foraging by (hawk)moths from plants that are adapted for pollination by other insects (see Haber and Frankie 1989; Martins and Johnson 2013). This study, hence, adds another record of moth pollination in *Erica*. The observed visits were associated with disrupted anther rings (a proxy for pollination in *Erica*) in roughly half the flowers examined, suggesting that moth visitation results in reasonably high pollination rates. Similar to the mechanism of pollen transfer in *E.cylindrica* (Van der Niet and Cozien 2022), pollen was found on the moths’ proboscises. Visits by *Helicoverpaarmigera* at Stettynsberg were somewhat unexpected, as the proboscis of this moth species is much shorter than the corolla tube of *E.infundibuliformis*. It is possible that this moth species, which is also the main pollinator of the moth-pollinated E.plukenetiisubsp.breviflora (Van der Niet et al. 2014b), is nevertheless able to access nectar that accumulates as droplets along most of the length of the corolla tube. A single bombyliid fly briefly visited *E.infundibuliformis* flowers during extensive daytime observation hours. Additionally, one individual of a *Moegistorhynchus* sp. was captured in the *E.infundibuliformis* population at Agtertafelberg. This fly not only carried *Erica* tetrads, but also had a tongue that closely matched the corolla tube of the local *E.infundibuliformis* plants in length. The pollen load and close morphological match with flower dimensions together suggest that *Moegistorhynchus* flies do occasionally visit the species, albeit at a lower rate than moths during the time that we observed plants. Low visitation rates of LPF to *Erica* species that are specialised for this pollinator group, with complete absence in some years, appears to be the norm (e.g. Newman and Johnson 2021; McCarren et al. 2023), raising the possibility that, in some years, flies may be more common visitors than observed during our study. However, regardless of whether and to what extent LPF contribute to pollination of *E.infundibuliformis*, evidence from this study shows that moths unambiguously contribute to pollination in this *Erica* species.

Moth visitation to *E.infundibuliformis* flowers is surprising because strong nocturnal floral scent is considered a key characteristic of moth-pollinated flowers (Faegri and van der Pijl 1979; Knudsen and Tollsten 1993; but see Martins and Johnson (2013)), whereas *E.infundibuliformis* flowers have not been described as scented to the human nose. Nevertheless, a weak scent was detectable when flowers were assessed in the field at the Stettynsberg study site, possibly owing to the elevated presence of linalool at this site in particular, and GC-MS analysis of *E.infundibuliformis* scent confirmed the presence of a scent bouquet that is richer in compounds than that of the closely related *E.cylindrica*, which is perceived as strongly scented by humans (Rebelo et al. 1985). Further, comparison of rates of scent emission showed that, at the time when moth pollinators are most active, during the evening hours, rates of scent emission are similar for both *E.infundibuliformis* and *E.cylindrica*. The main difference between the scent of the two *Erica* species is that *E.cylindrica* flowers emit aromatic compounds, whereas the scent of *E.infundibuliformis* flowers is dominated by monoterpenes, such as ocimene. In analyses of human perception of volatiles, based on gas chromatography coupled with olfactometry, (E)-ocimene is often reported as an active compound (Lee et al. 2011; Zhao et al. 2022), but it is sometimes described by humans as grass-like and perhaps, therefore, not considered a typical floral volatile. Together, these results reinforce the need to objectively quantify floral traits when assigning pollination syndromes to plant species.

Although the function of scent for moth attraction in *E.infundibuliformis* was not established experimentally, some evidence supports the idea. Despite the scent of *E.infundibuliformis* not conforming to a typical bouquet associated with moth pollination, hawkmoth (including the species visiting *E.infundibuliformis*) antennae respond to (E)-ocimene (Shuttleworth and Johnson 2022) and high emission rates of the two stereoisomers of ocimene in an orchid were found to coincide with the peak activity of hawkmoths (Steen et al. 2019), suggesting a functional role for monoterpenes in moth attraction. Additionally, the objective absence of scent recorded by GC-MS analysis of flowers of two *Erica* species pollinated exclusively during the day by LPF (McCarren et al. 2023) further supports a functional role of scent emission for pollinator attraction in *E.infundibuliformis*. Although lower scent emission during the evening than during daylight hours contradicts a typical moth syndrome (Jürgens et al. 2014; Balducci et al. 2020; Powers et al. 2020), this pattern has also been found in other moth-pollinated plant species (Van der Niet et al. 2015). An interesting analogous case of interspecific variation in floral scent composition, as found in this study between *E.infundibuliformis* and *E.cylindrica*, occurs in two closely-related *Zaluzianskya* species, although these species do differ in the pollination system: *Z.natalensis*, with a bouquet dominated by aromatics, is pollinated by hawkmoths, whereas *Z.microsiphon*, dominated by monoterpenes, is pollinated by long-proboscid nemestrinid flies (Campbell et al. 2016). However, unlike in *Erica*, in this *Zaluzianskya* species pair, the flowers of the fly-pollinated species close during the evening and experimental manipulations and bioassays showed that flower visitation by hawkmoths was determined by flower orientation rather than by scent composition.

Flowers of both moth-pollinated *Erica* species studied here were found to be predominantly facing upwards, which is highly unusual in the genus (Van der Niet and Cozien 2022). In other systems, the upward-facing orientation of flowers has been shown to be important for pollination by hawkmoths (Fulton and Hodges 1999; Campbell et al. 2016). Upward-facing flowers should, therefore, not be considered exclusively associated with nemestrinid fly pollination in *Erica* (see McCarren et al. 2022; McCarren et al. 2023), as this study further confirms its association with pollination by moths (and hawkmoths in particular) in *Erica* (see also Van der Niet and Cozien (2022)).

Variation in corolla tube length is often associated with covariation with pollinator morphology as the match may be important for effective pollen transfer (e.g. Van der Niet et al. 2014b; Newman and Johnson 2021). In this study, corolla tube length was found to differ amongst the three *Erica* populations, with the longest corolla tubes found for *E.infundibuliformis* at Agtertafelberg and the shortest in *E.cylindrica*, albeit only marginally shorter than *E.infundibuliformis* at Stettynsberg. However, there was no consistent covariation or trait matching between pollinator proboscis length and floral tube length amongst populations or species; many legitimate flower visitors had proboscides that exceeded the floral tube in length (Fig. 6). This general absence of covariation is not unexpected for interactions in which pollination precision is low, as is the case for both the study species of *Erica*, when anthers of the flowers do not protrude beyond the floral tube and pollen is mostly placed quite imprecisely along the moth proboscis (also see Johnson 2024). It is possible that shorter floral tubes at Stettynsberg might reflect higher rates of visitation by relatively short-tongued settling moths versus predominant visitation by longer-tongued hawkmoths at Agtertafelberg, but more pollinator observations are required to firmly establish any difference in moth species composition between the two sites.

The two studied *Erica* species differ in colour as perceived by humans and differences were confirmed by objectively measured reflectance spectra of the two species. In both species, brightness of the corolla tube is lower than for the petals, but in both Agtertafelberg and Stettynsberg, reflectance of petal lobes of *E.infundibuliformis* is approximately twice that recorded for *E.cylindrica* (see Van der Niet and Cozien (2022)). Flowers of *E.cylindrica* have maximum reflectance between the 500 and 700 nm region, mainly in the human-visible part of the spectrum, whereas flowers of *E.infundibuliformis* also strongly reflect light in the UV range of the spectrum, with a steep increase in the UV region around 350 nm and consistently high maximum reflectance from 400 to 700 nm. The flower colour is perhaps the most puzzling trait in association with moth-pollination, as moth-pollinated flowers usually do not exhibit this steep increase in reflectance in the UV range (e.g. Johnson and Raguso (2005)), which is, instead, strongly characteristic for other fly-pollinated *Erica* species (Lombardi et al. 2021; Newman and Johnson 2021; Pauw 2022; McCarren et al. 2023). The colour of the floral tube, which does not reflect light in the UV region may be particularly visible to many Hymenoptera and, therefore, explain their attraction to this flower part for nectar consumption (Chittka et al. 1994). However, they may also be attracted by the scent or their presence may be driven by local distribution, since they were not observed at Stettynsberg.

**Figure 8.**
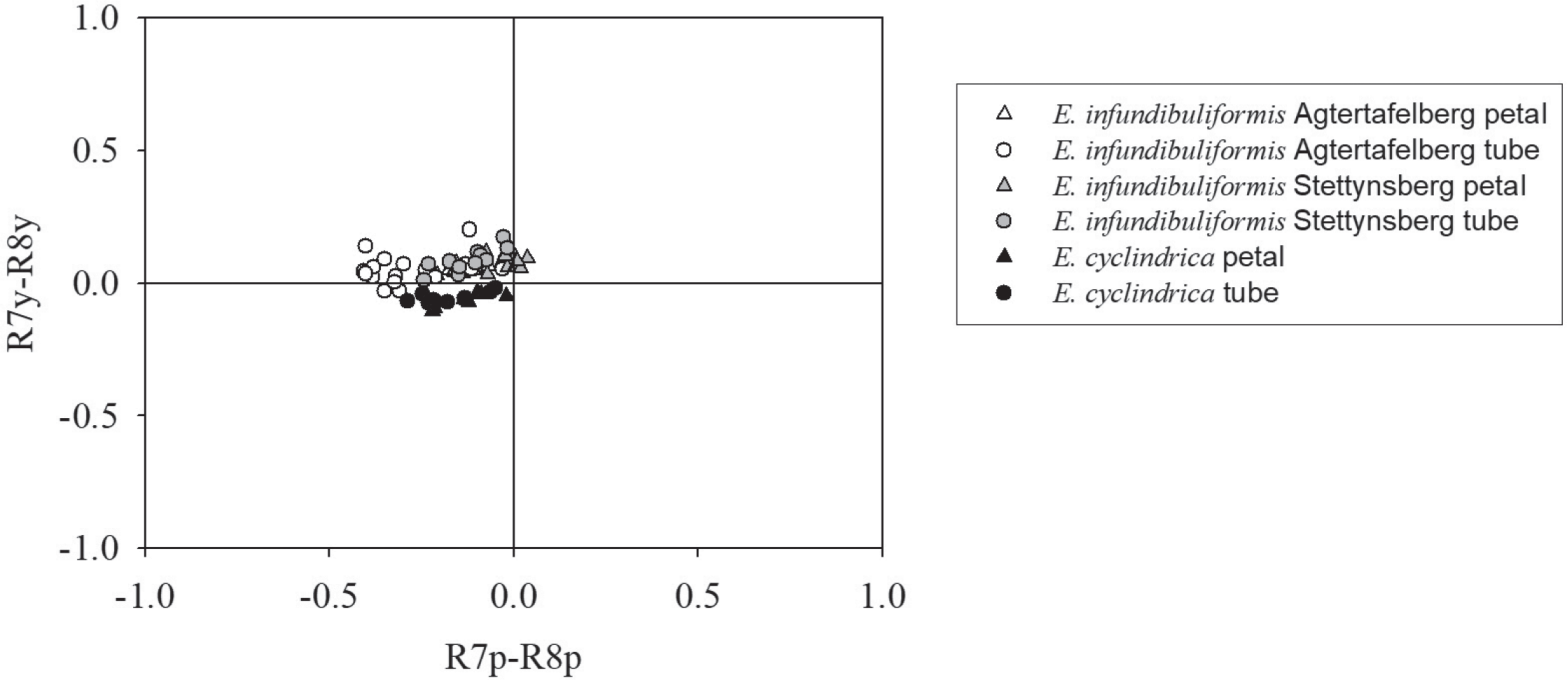
Loci of colours of petals and corolla tubes for flowers of *Ericainfundibuliformis* from Aftertafelberg and Stettynsberg and for *E.cylindrica*, plotted in the fly vision colour space of Troje (1993). Loci of most, but not all spectra of both petals and floral tubes of flowers from both populations of *E.infundibuliformis* fell within a single quadrant in the fly vision colour space; spectra of both petals and floral tube surfaces of *E.cylindrica* fell into a different quadrant, suggesting that neither species has within-flower contrast for flies, but that flies may perceive flowers of the two species differently.

The apparent contradiction between observed pollinators and floral traits of *E.infundibuliformis* raises the question for what kind of pollinator the species is adapted. Although the possibility that LPF pollination was underestimated cannot be excluded, effective pollination by moths is unambiguous. Some of the quantified traits, such as the presence of floral scent, suggest a functional role in moth pollination, but not fly pollination. Flower colour, on the other hand, was more typical for LPF-pollinated ericas (McCarren et al. 2021). One possibility is that, for these two traits (scent and colour) in this system, there are not strong trade-offs associated with attraction of moths versus flies, as has been suggested for other specialised pollination systems (Aigner 2001) and shown for particular floral traits in other plant species (Muchhala 2007; Peakall et al. 2010). Cases where trade-offs have been demonstrated involved roles of highly specific sex pheromones for pollinator attraction or morphological mismatches. It is possible that the presence of certain scent compounds may not compromise fly pollination (Campbell et al. 2016), whereas flower colour may be less critical for nocturnal moth pollination. The pollination system of *E.infundibuliformis* may, thus, represent a combination of traits in which an absence of trade-offs facilitates a bimodal pollination system, which has been observed in several species of *Tritoniopsis* (Manning and Goldblatt 2005) and in *Proteapunctata* (Johnson et al. 2012). Indeed, visitation by hawkmoths and LPF has been demonstrated in other long-tubed plants in South Africa that are seemingly unscented to the human nose, such as *Satyriumhallackii* (Johnson 1997). Such bimodal pollination systems may be particularly important if visitation by one of the pollinator groups is unpredictable, as in this case may apply to LPF. Interpretation of traits of *E.infundibuliformis* in the context of bimodal pollination rather than as exclusive adaptations for moth pollination is also consistent with the observed trait divergence relative to *E.cylindrica*. If both species were specialised for moth pollination, the observed differences in floral traits, most strikingly flower colour, which has shifted between quadrants in a model of fly vision (Fig. 8), would be particularly puzzling. Further studies, including temporal selective pollinator exclusion experiments to assess the potential role of diurnal versus nocturnal pollinators (e.g. Wenzell et al. 2024) and phylogenetic analyses that can be used to determine the evolutionary direction of floral trait divergence, are needed to obtain a better understanding of the evolution of pollination systems (Van der Niet et al. 2014b).

This study adds to a number of cases in which syndrome-based hypotheses were contradicted by empirical observations (e.g. de Merxem et al. 2009; Cozien et al. 2019; Castañeda-Zárate et al. 2021). In the study system examined here, the combination of rarity of hawkmoth pollination in the CFR, in general and in *Erica*, in particular, in combination with the inadequacy of human perception for identification of floral scent, were probably the main reasons why moth pollination was not correctly predicted. It is also possible that the trait combination in *E.infundibuliformis* represents a syndrome indicative of bimodal pollination by flies and moths, but data are currently inadequate to confirm or refute this idea. Our findings again underline the importance of verifying pollination systems predicted by syndromes with empirical pollinator observations (Van der Niet 2021). Results also indicate that hawkmoth pollination, which has now been documented at multiple sites and years and during different seasons, may be more common in the CFR than previously suggested (Johnson 1997) and emphasise that, despite the associated challenges, pollinator observations during evening hours should be considered a critical component in any pollination study of plant species with long-tubed flowers and to characterise the pollination systems of any region (cf. Cai et al. 2024).

## ﻿Additional information

### Author ORCIDs

Timotheüs van der Niet https://orcid.org/0000-0002-5250-8995

Ruth J. Cozien https://orcid.org/0000-0001-6800-5383

### Data availability

All of the data that support the findings of this study are available in the main text or Supplementary Information.

## Supplementary materials

Supplementary material 1 Mass spectra of unknown compounds Data type pdf Explanation note Mass spectra of unknown compounds that were found across an entire batch or in samples across multiple batches in the scent of *Ericainfundibuliformis*. Two compounds for which identification was somewhat ambiguous are also included. https://binary.pensoft.net/file/1126453This dataset is made available under the Open Database License (http://opendatacommons.org/licenses/odbl/1.0/). The Open Database License (ODbL) is a license agreement intended to allow users to freely share, modify, and use this Dataset while maintaining this same freedom for others, provided that the original source and author(s) are credited.Timotheüs van der Niet, Ruth Cozien
