# Supplementary material for: ﻿Evidence for moth pollination in a rhinomyiophilous Erica species from the Cape Floristic Region of South Africa
Source: PhytoKeys. 2024 Sep 2;246:43–70. doi: 10.3897/phytokeys.246.126310 (PMC11384911; doi:10.3897/phytokeys.246.126310)
Supplement: Supplementary material 1 — Mass spectra of unknown compounds [file phytokeys-246-043_article-126310__-s001.pdf]

1121

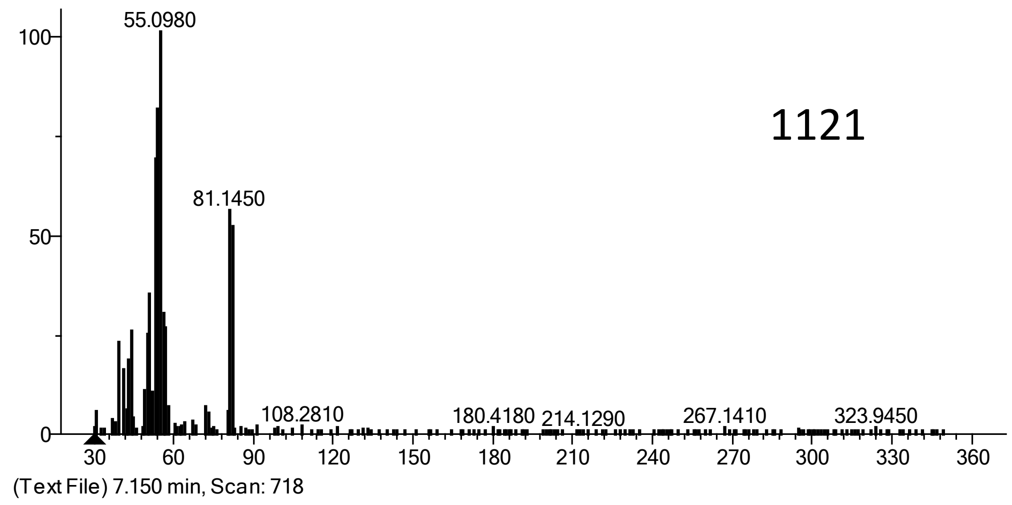

1358

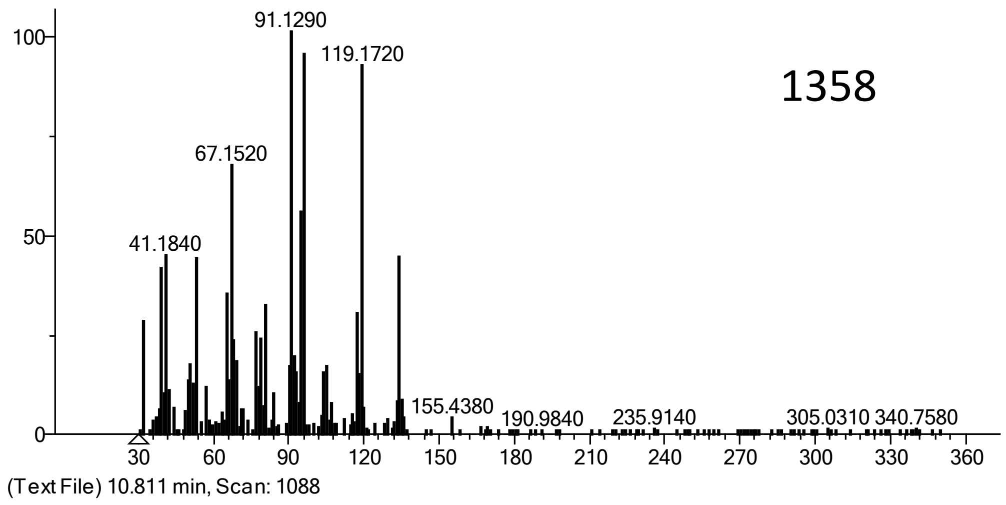

1464

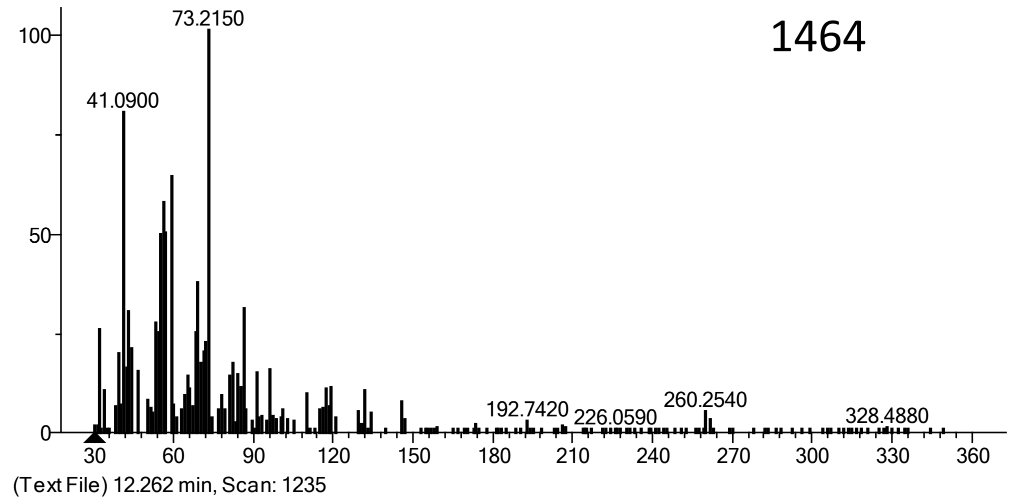

1490

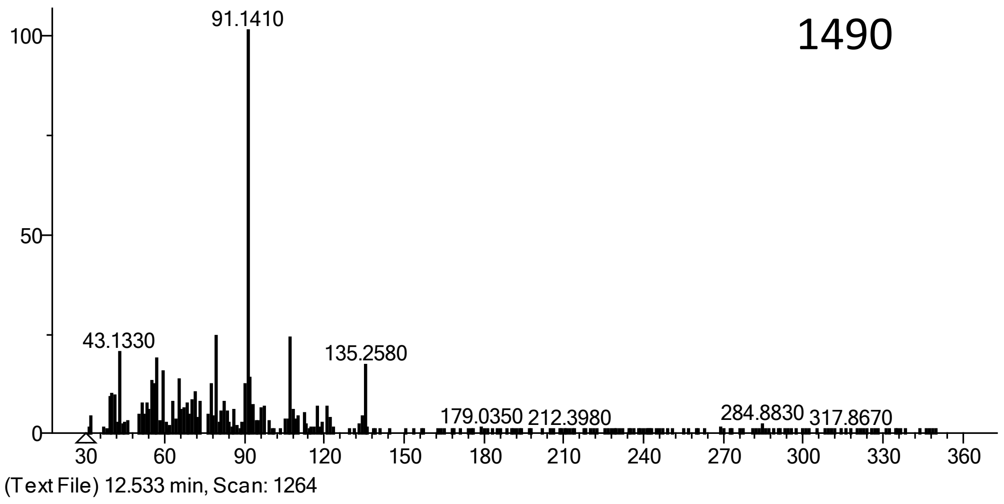

1511

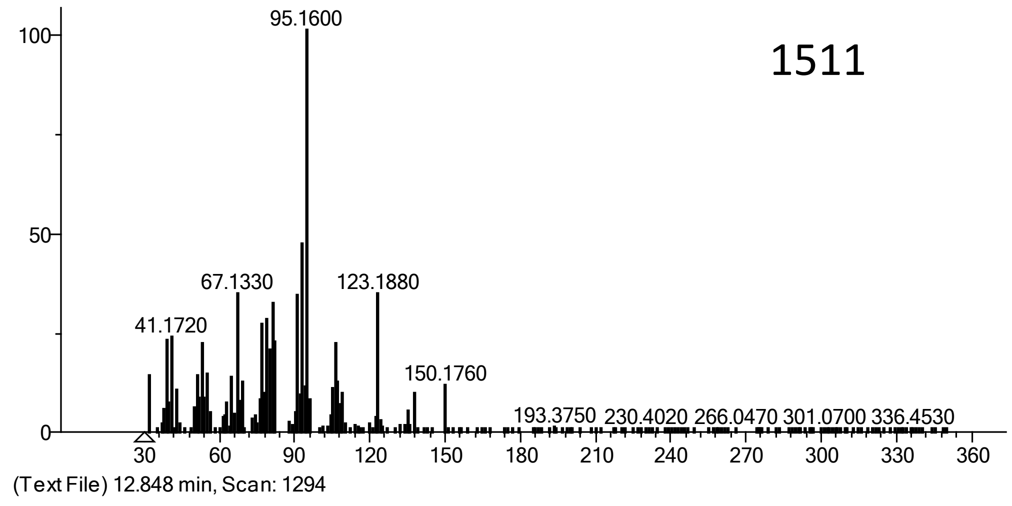

1524

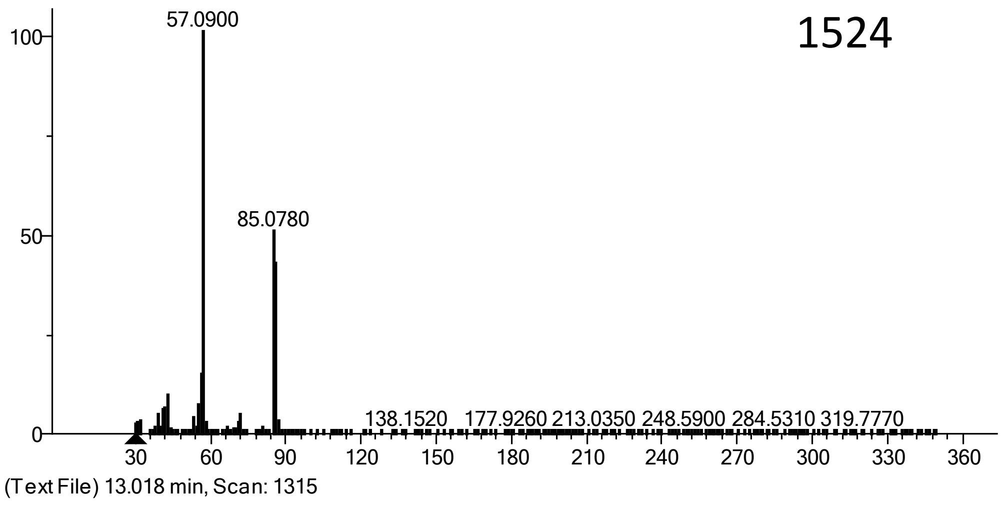

1542
Cinerone
Listed under monoterpenes

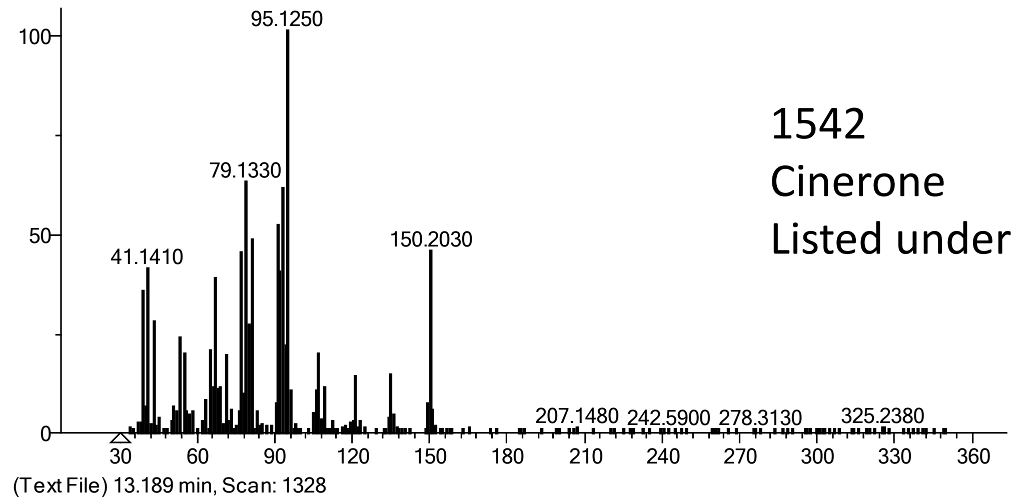

1650

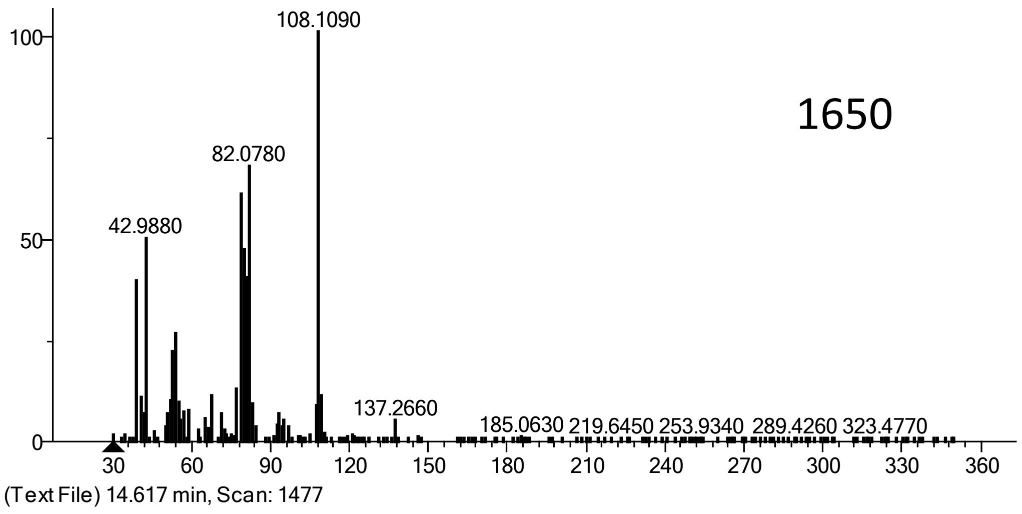

1700

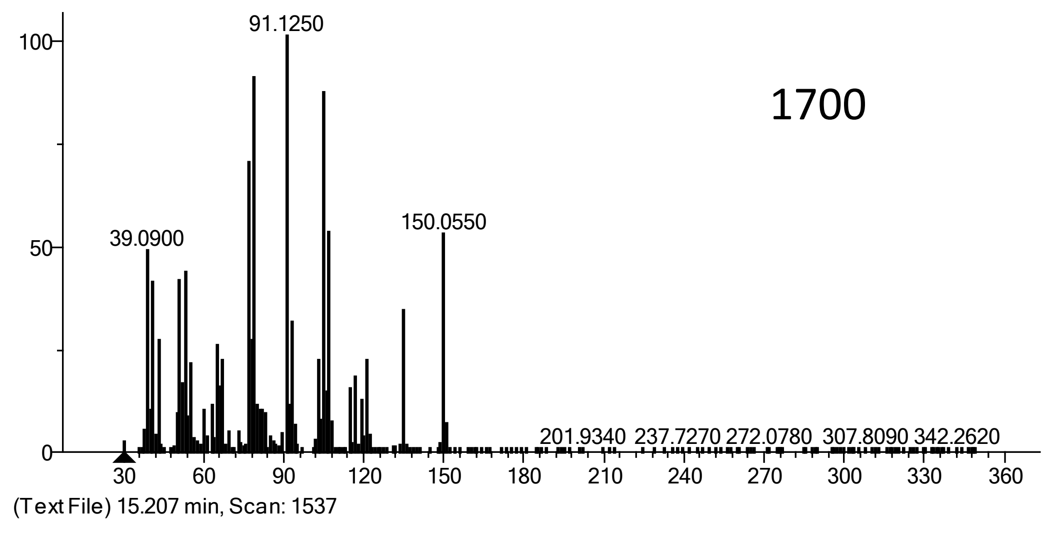

1771

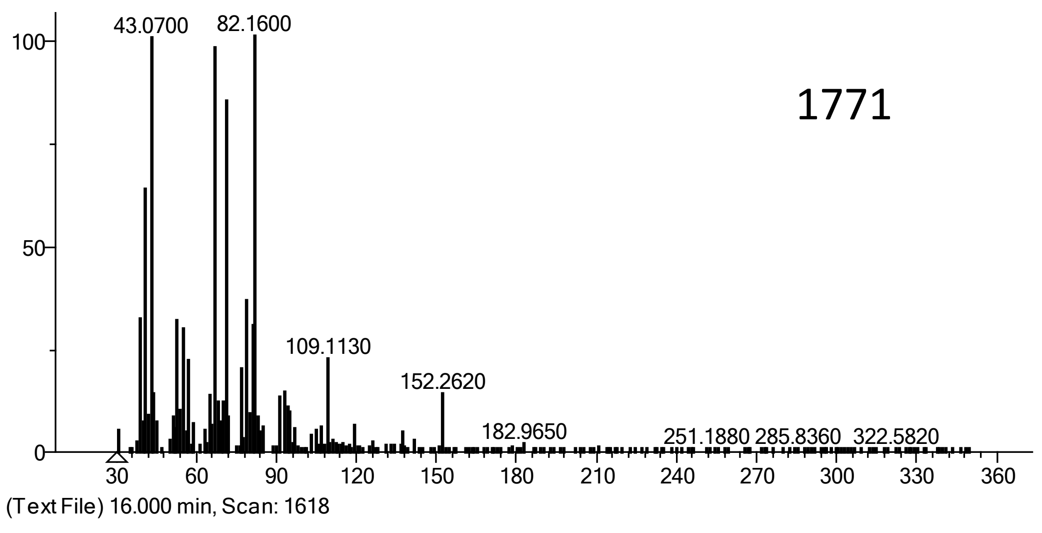

1844

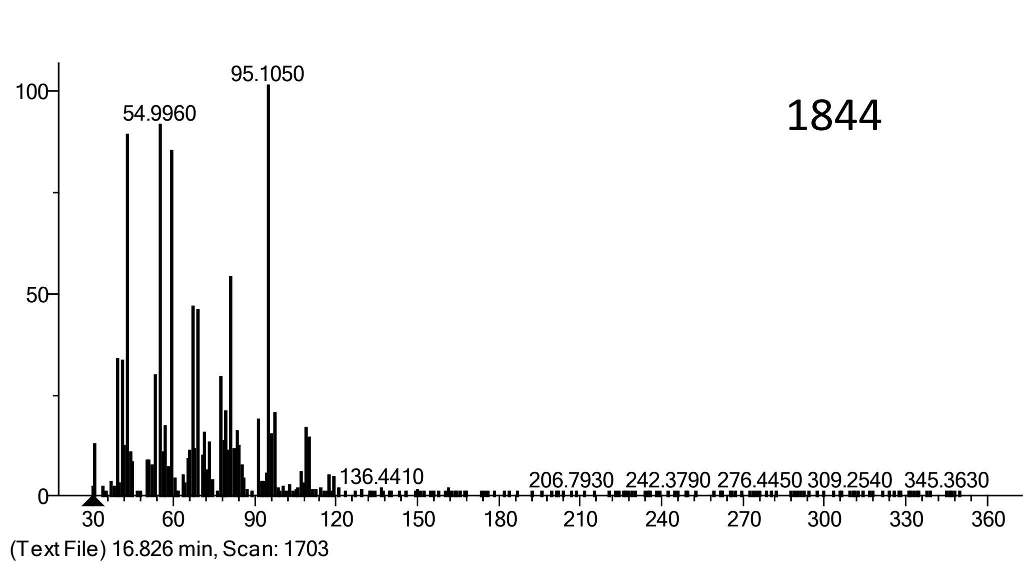

1848

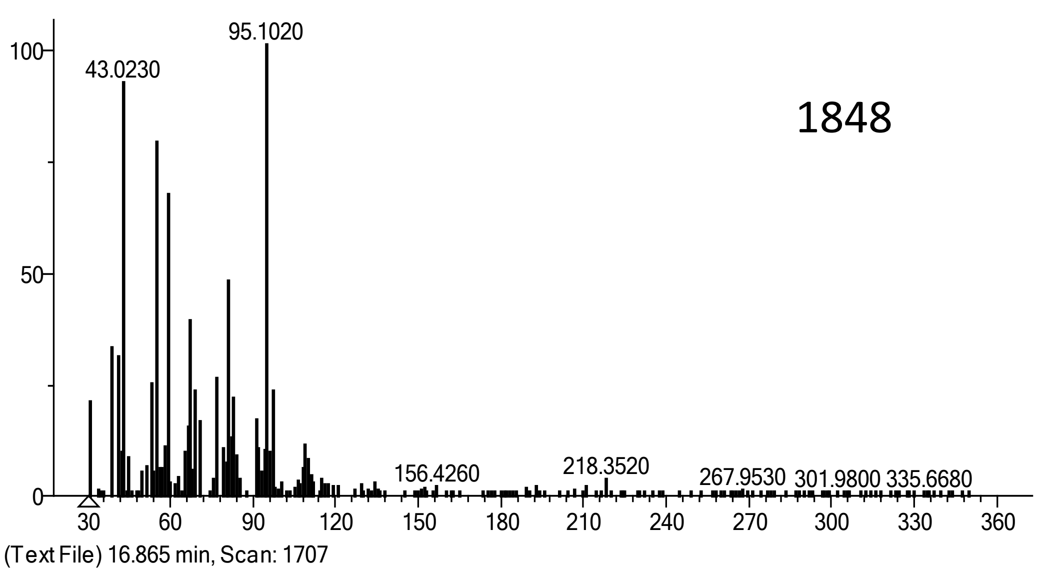

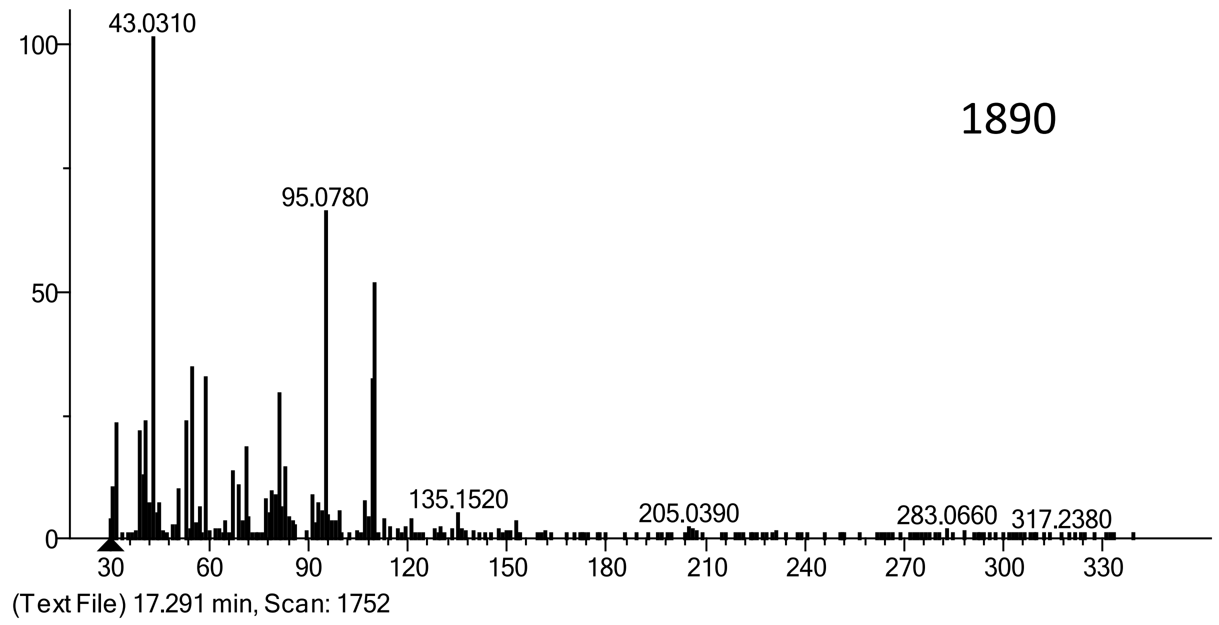
1890
43.0310
95.0780
135.1520
205.0390
283.0660
317.2380
(Text File) 17.291 min, Scan: 1752

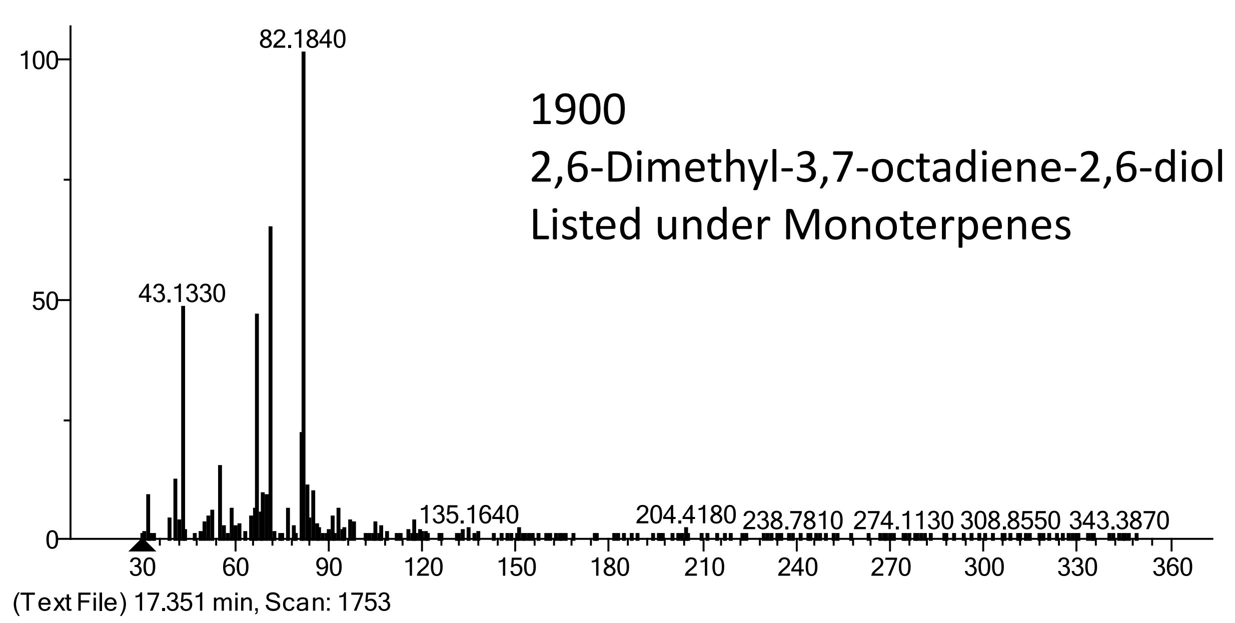
1900
2,6-Dimethyl-3,7-octadiene-2,6-diol
Listed under Monoterpenes
82.1840
43.1330
135.1640
204.4180
238.7810
274.1130
308.8550
343.3870
(Text File) 17.351 min, Scan: 1753

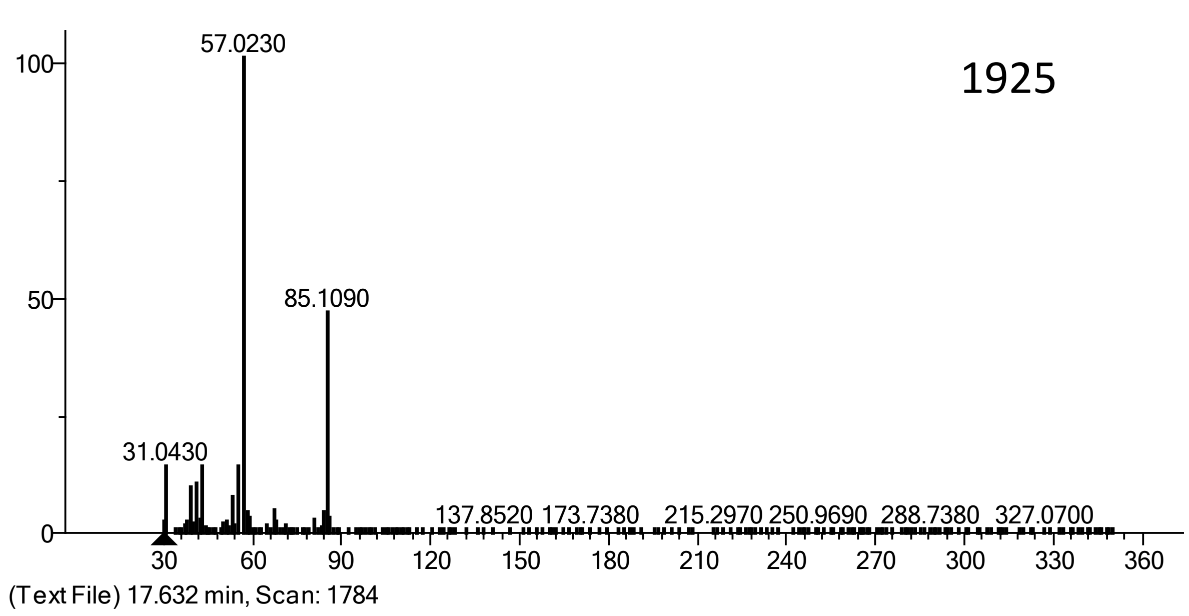
1925
57.0230
85.1090
31.0430
137.8520
173.7380
215.2970
250.9690
288.7380
327.0700
(Text File) 17.632 min, Scan: 1784

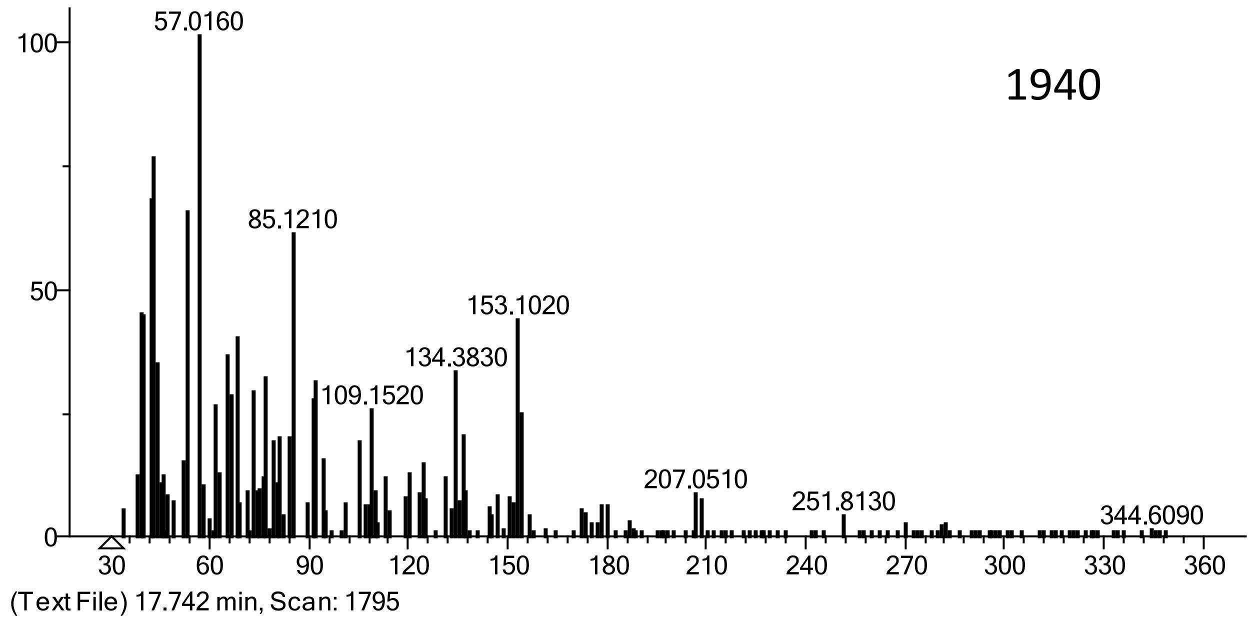
1940
57.0160
85.1210
153.1020
134.3830
109.1520
207.0510
251.8130
344.6090
(Text File) 17.742 min, Scan: 1795

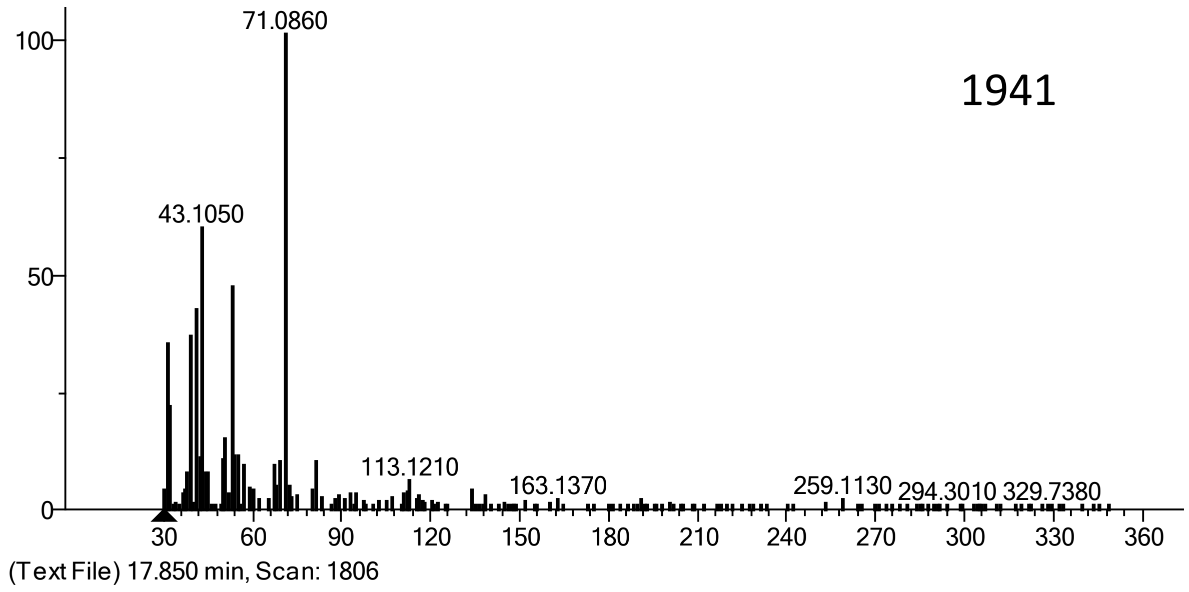
1941
71.0860
43.1050
113.1210
163.1370
259.1130
294.3010
329.7380
(Text File) 17.850 min, Scan: 1806

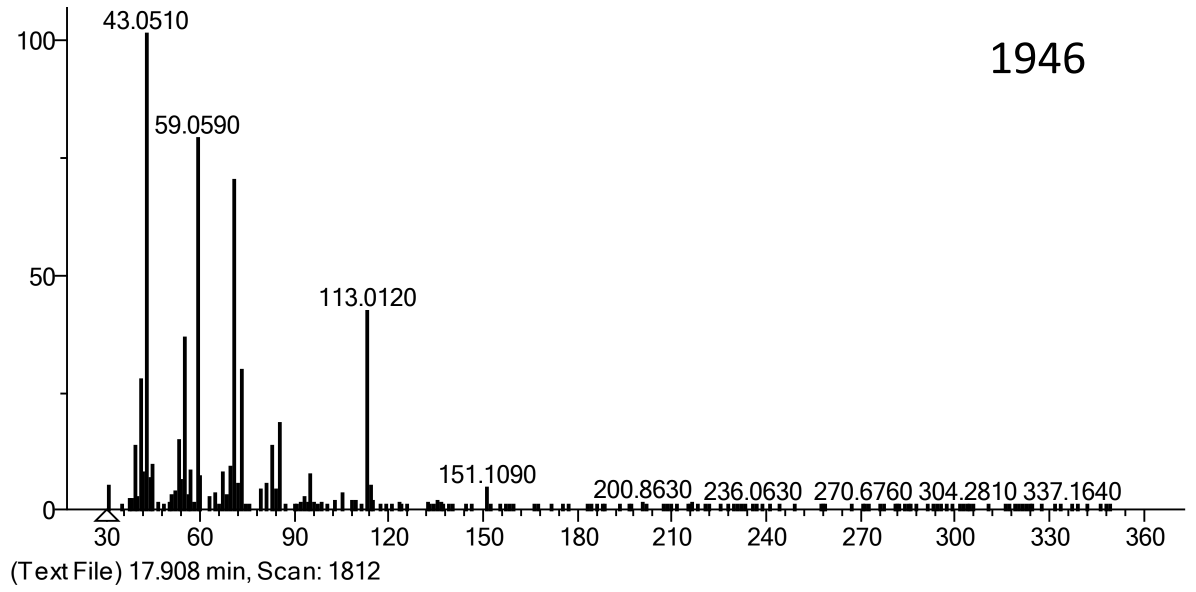
1946
43.0510
59.0590
113.0120
151.1090
200.8630
236.0630
270.6760
304.2810
337.1640
(Text File) 17.908 min, Scan: 1812

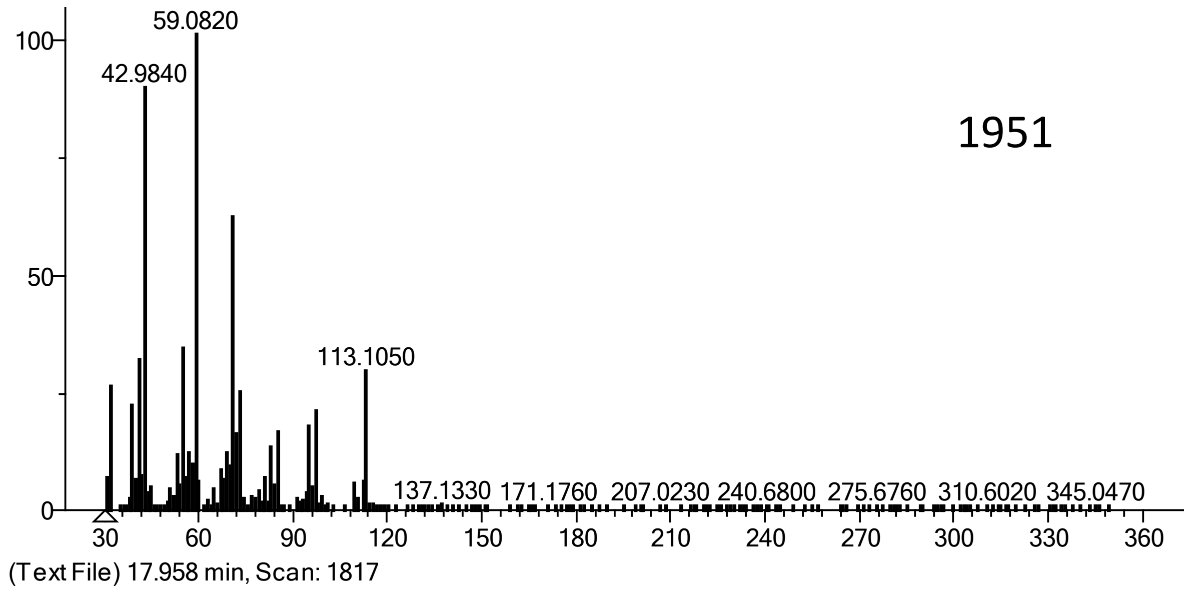
1951
59.0820
42.9840
113.1050
137.1330
171.1760
207.0230
240.6800
275.6760
310.6020
345.0470
(Text File) 17.958 min, Scan: 1817

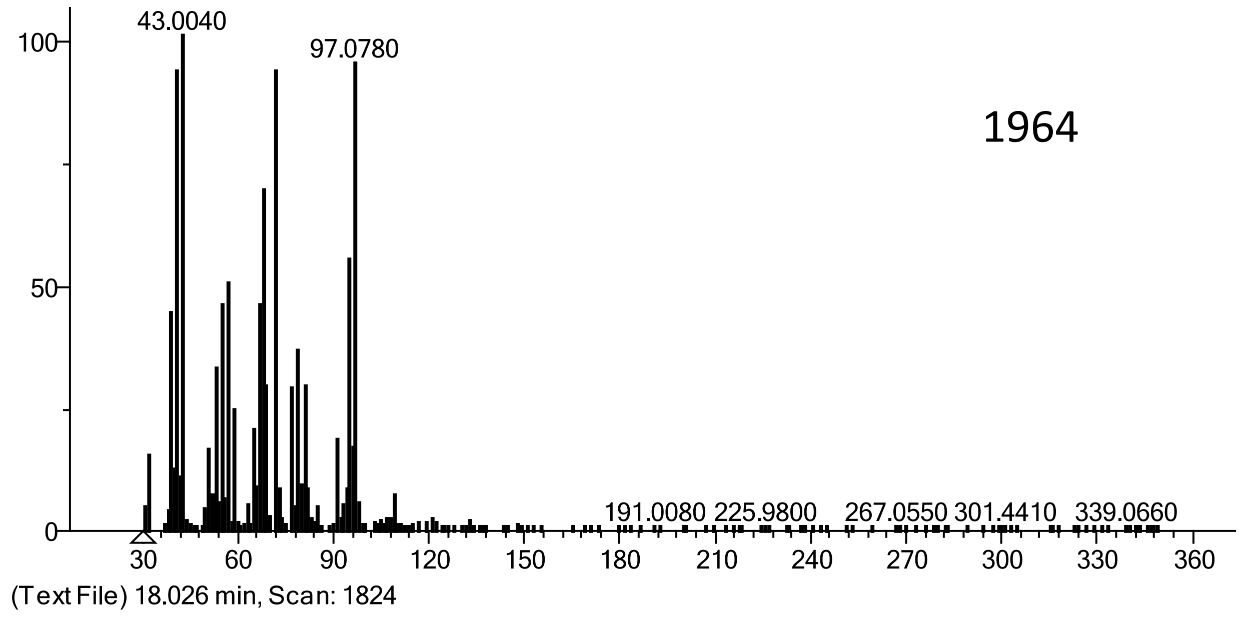
1964
43.0040
97.0780
191.0080
225.9800
267.0550
301.4410
339.0660
(Text File) 18.026 min, Scan: 1824

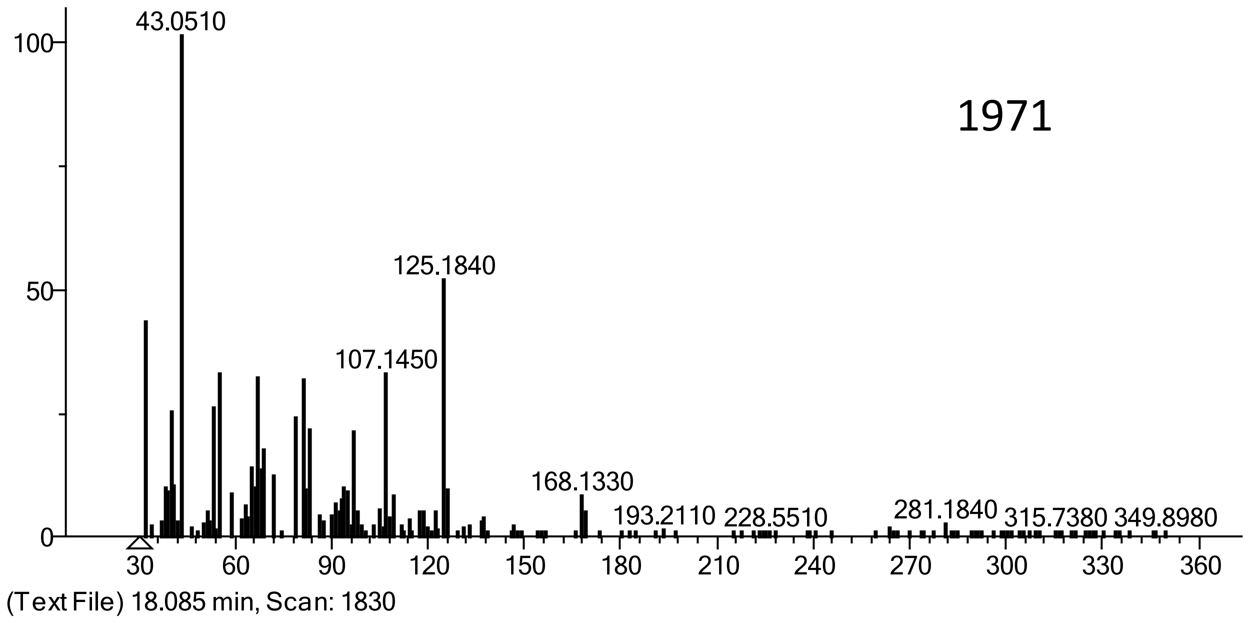
1971
43.0510
125.1840
107.1450
168.1330
193.2110
228.5510
281.1840
315.7380
349.8980
(Text File) 18.085 min, Scan: 1830

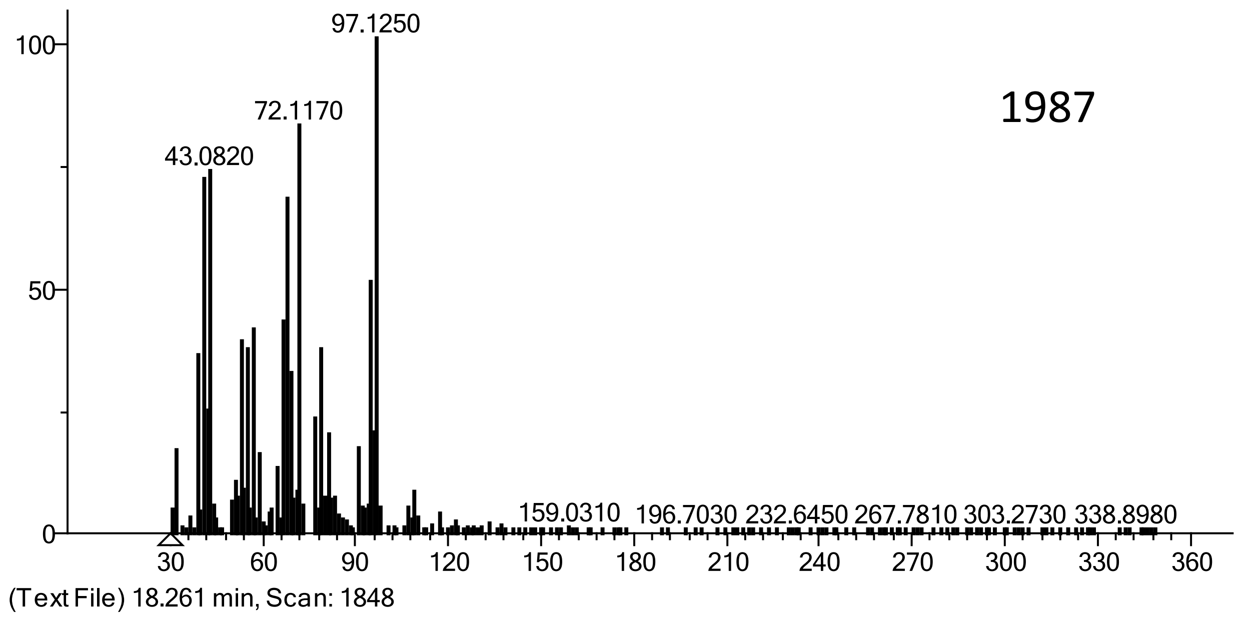
1987
97.1250
72.1170
43.0820
159.0310
196.7030
232.6450
267.7810
303.2730
338.8980
(Text File) 18.261 min, Scan: 1848

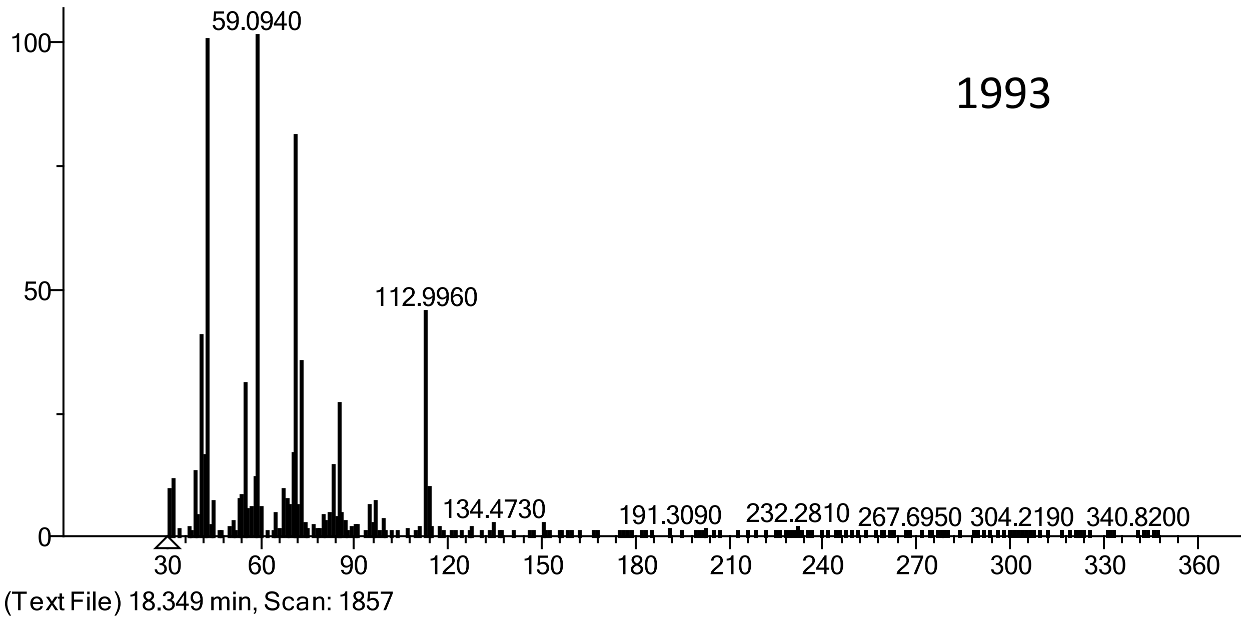
1993
59.0940
112.9960
134.4730
191.3090
232.2810
267.6950
304.2190
340.8200
(Text File) 18.349 min, Scan: 1857

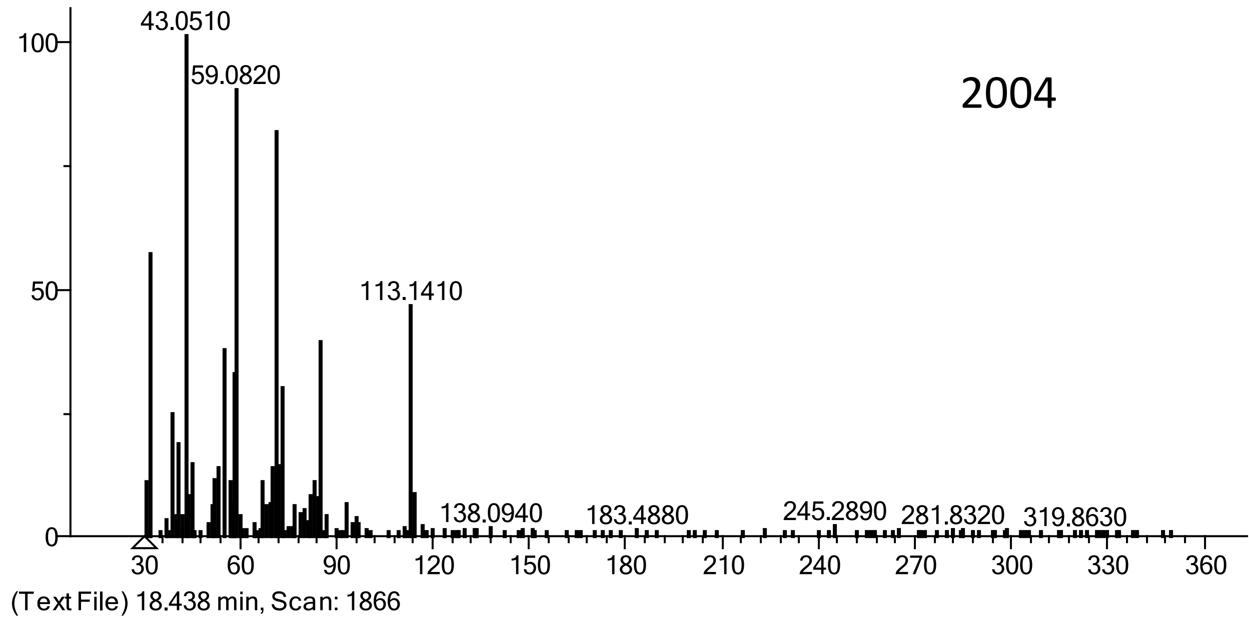
2004
43.0510
59.0820
113.1410
138.0940
183.4880
245.2890
281.8320
319.8630
(Text File) 18.438 min, Scan: 1866

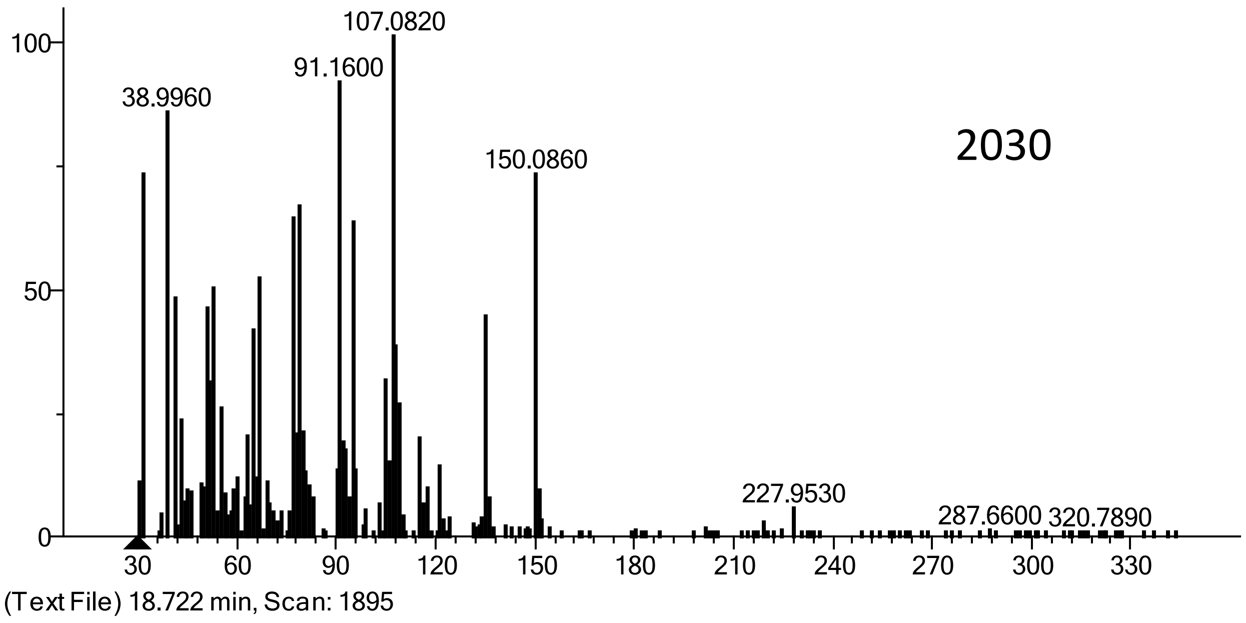
2030
38.9960
91.1600
107.0820
150.0860
227.9530
287.6600
320.7890
(Text File) 18.722 min, Scan: 1895

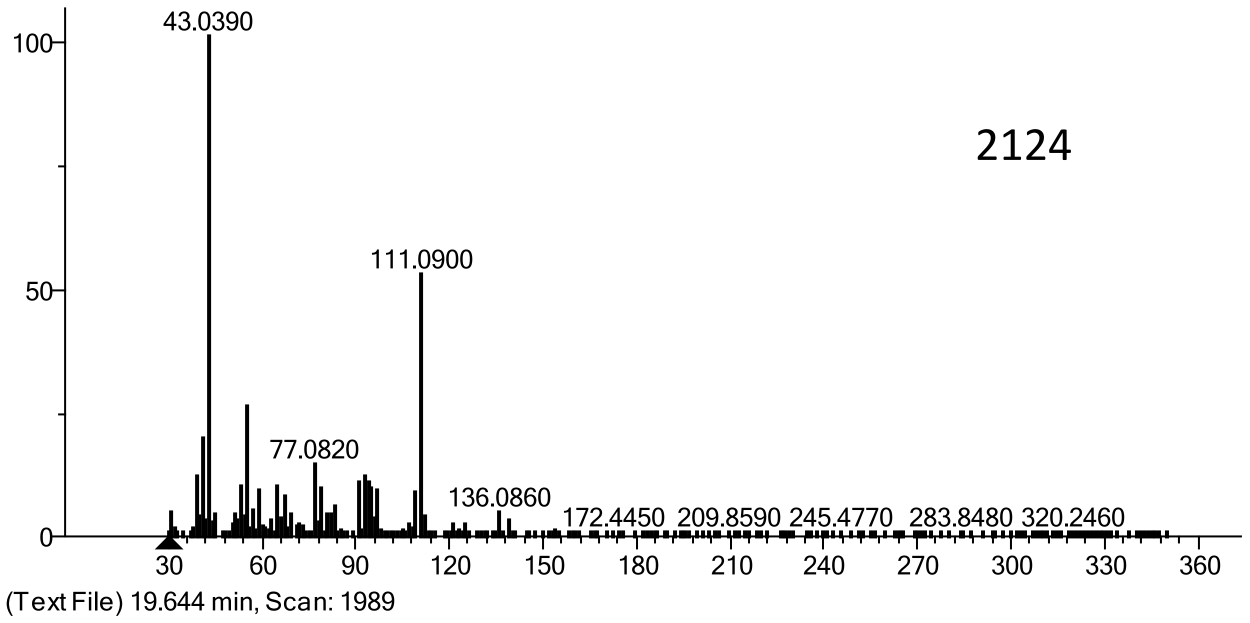
2124
43.0390
111.0900
77.0820
136.0860
172.4450
209.8590
245.4770
283.8480
320.2460
(Text File) 19.644 min, Scan: 1989

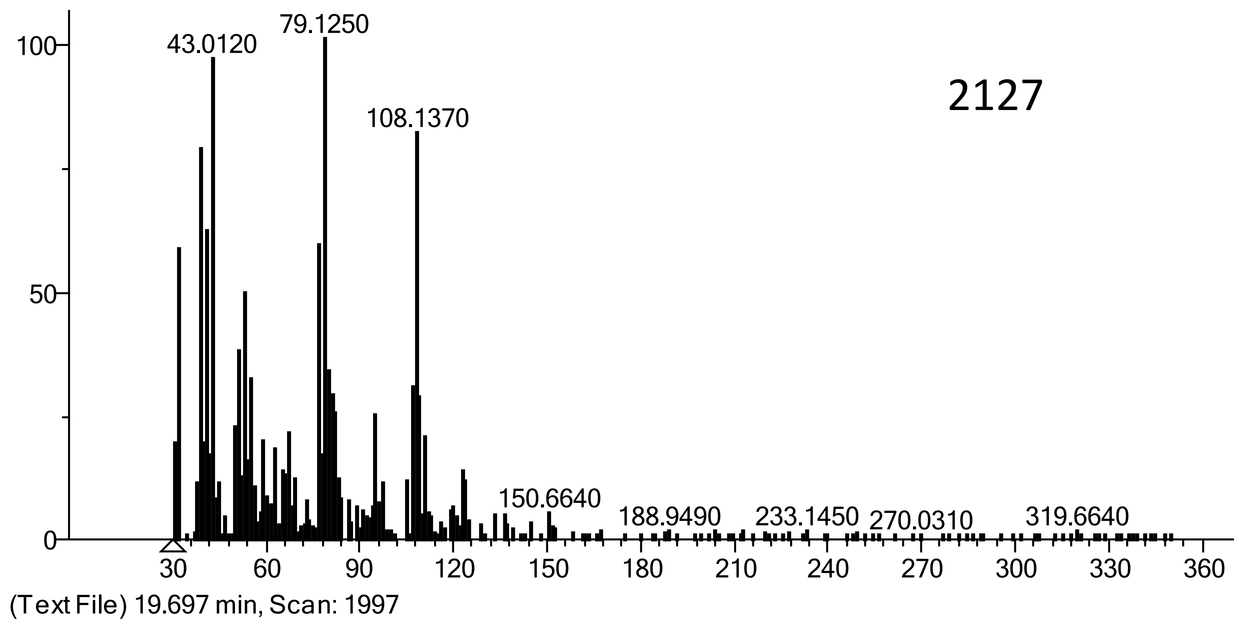
2127
43.0120
79.1250
108.1370
150.6640
188.9490
233.1450
270.0310
319.6640
(Text File) 19.697 min, Scan: 1997

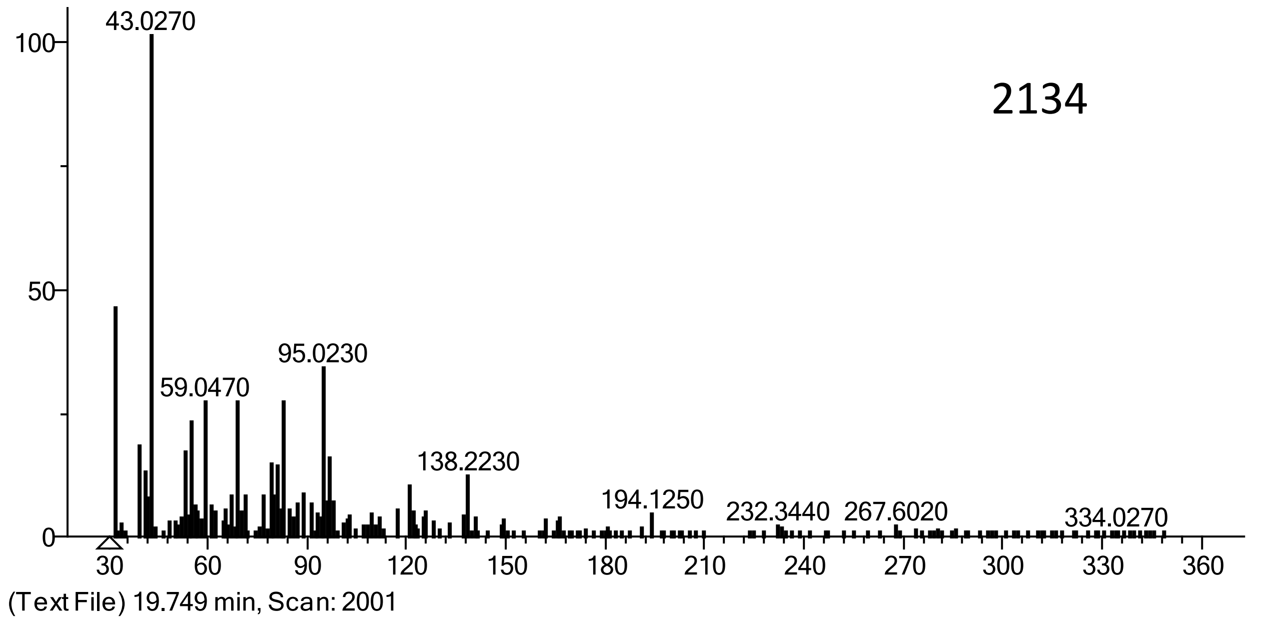
2134
43.0270
59.0470
95.0230
138.2230
194.1250
232.3440
267.6020
334.0270
(Text File) 19.749 min, Scan: 2001

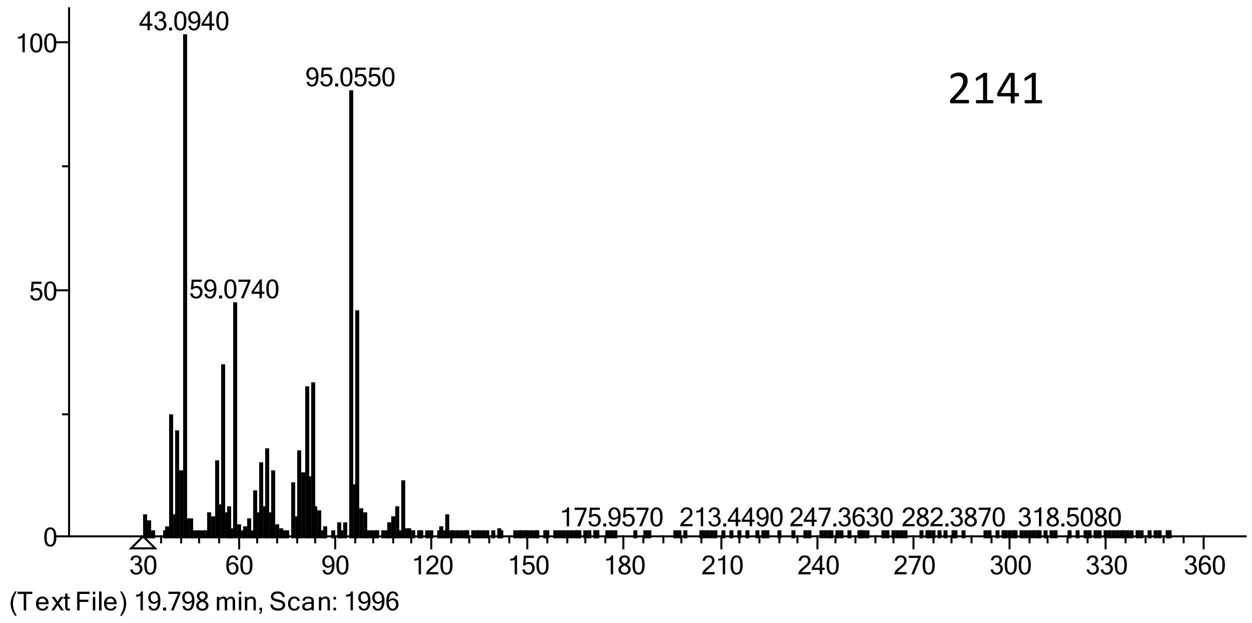
2141
43.0940
95.0550
59.0740
175.9570
213.4490
247.3630
282.3870
318.5080
(Text File) 19.798 min, Scan: 1996

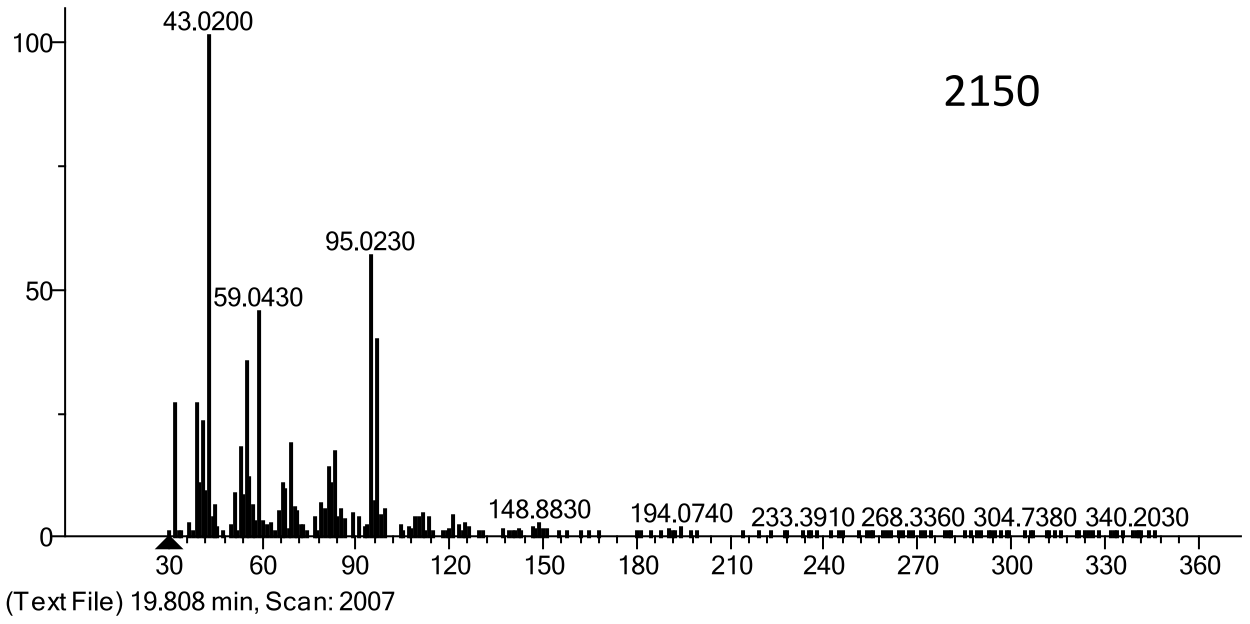
2150
43.0200
95.0230
59.0430
148.8830
194.0740
233.3910
268.3360
304.7380
340.2030
(Text File) 19.808 min, Scan: 2007

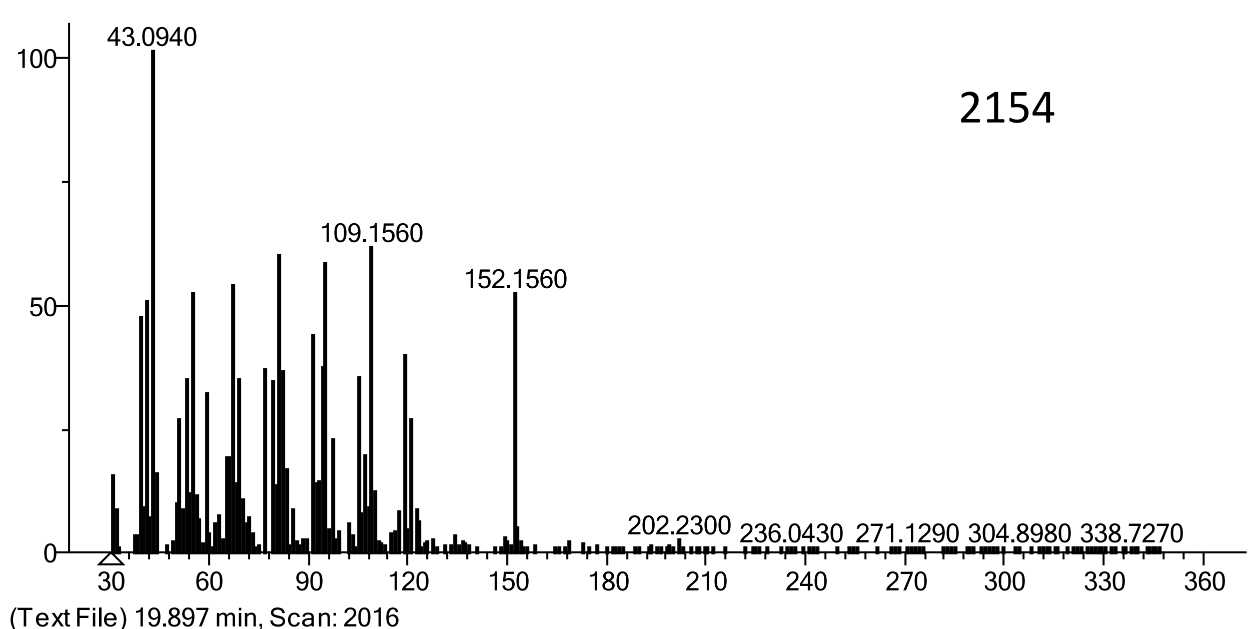
2154
43.0940
109.1560
152.1560
202.2300
236.0430
271.1290
304.8980
338.7270
(Text File) 19.897 min, Scan: 2016
